# Profiling of Primary Metabolites and Volatiles in Apricot (*Prunus armeniaca* L.) Seed Kernels and Fruits in the Context of Its Different Cultivars and Soil Type as Analyzed Using Chemometric Tools

**DOI:** 10.3390/foods11091339

**Published:** 2022-05-04

**Authors:** Mohamed A. Farag, Nehal S. Ramadan, Mohamed Shorbagi, Nermeen Farag, Haidy A. Gad

**Affiliations:** 1 Pharmacognosy Department, Faculty of Pharmacy, Cairo University, Cairo 11562, Egypt; nermeen.farag@pharma.cu.edu.eg; 2 Chemistry of Tanning Materials and Leather Technology Department, National Research Centre, Dokki, Cairo 12622, Egypt; nehalsameh111@gmail.com; 3 Department of Special Chemistry, Faculty of Science, Benha University, Benha 13511, Egypt; mohamedshorbagi1@gmail.com; 4 Pharmacognosy Department, Faculty of Pharmacy, Ain Shams University, Cairo 11566, Egypt; haidygad@pharma.asu.edu.eg

**Keywords:** apricot, GC–MS, metabolomics, *Prunus armeniaca* L., solid-phase micro-extraction (SPME), volatile profiling

## Abstract

The goal of this study was to assess nutrient primary metabolites and aroma determinants in *Prunus armeniaca* L. fruits and seed kernels grown in Egypt represented by its different cultivars and agricultural conditions i.e., two different soil types (muddy versus sandy). Two techniques were employed to assess non-volatile and volatile metabolites using gas chromatography mass-spectrometry (GC-MS) post silylation, and headspace solid-phase micro-extraction (HS-SPME) coupled GC-MS, respectively. A total of 36 peaks belonging to sugars, fatty acids/esters and organic acids were identified by GC–MS in various apricot fruits and seed kernels cultivars. Glucose and sucrose were enriched in apricot fruits compared to the seed kernels. A total of 70 volatiles were identified, with lactones, alcohols and esters representing the main classes of apricot volatiles accounting for its discrete aroma. (*E*)-Anethole, β-ionone, γ-decanolactone and methyl palmitate were the major peaks contributing to the discrimination between various fruit cultivars and providing novel insight on apricot metabolome.

## 1. Introduction

The increasing need to assess the nutritive value and safety of food mandates the implementation of advanced chemical analysis methodologies [1] that can determine its quality considering its complex chemical composition. Geographical origin, agricultural practices, growing conditions and genotype in addition to the complexity of food matrices are known to affect levels of its bioactive compounds [2].

Apricot *Prunus armeniaca* L., Family: (Rosaceae) also known as *Armeniaca vulgaris* L. or cultivated apricot, is a popular edible drupe fruit that is closely related to peaches or plums. With a total world production of ca. 4 million tons, apricots are extensively grown worldwide, but are mainly cultivated in Mediterranean climates [3,4]. The global trend of consumers’ purchasing preferences switched to distinctive fruit crops with higher nutritional content and a pleasant flavor and to account for the rapid development and increase in cultivation of stone fruit crops *videlicet* (*viz.)* apricots. Moreover, apricot distinct aroma, delicious taste, high nutritive value, multiple uses pose it to be produced even more widely and to contribute more to the world’s fruit production [4]. Asides from its rich nutritive value, apricot fruit is reported for the treatment of liver disorders [5], hemorrhages, piles, infertility, eye inflammation, respiratory and digestive diseases in addition to its anti-diarrheic, anti-pyretic, emetic, anthelmintic and aphrodisiac proprerties [6,7]. Health effects reported for seeds include hepatoprotective, analgesic, anthelmintic, anti-asthmatic, antispasmodic, demulcent, emollient and treatment of lung or liver cancer [5,8] and externally for face care [9]. Whereas, tree root and bark decoctions are used to soothe inflamed and irritated skin conditions and in the treatment of asthma, coughs, acute or chronic bronchitis, poisoning and constipation [6].

Egypt as a Mediterranean country is a major producer of apricot in the region with production of around 98,295 tonns in 2019 according to FAO stats [10] represented by various cultivars cultivated in Egypt *viz.* Balady, Amar, and Hamawy, with continuous introduction of several new varieties by the Ministry of Agriculture and private sector in order to prolong its marketing period [11], considering its short shelf life. Although the typical Egyptian soil composition is mudy from the Nile, in the fringes of the Nile Delta and Valley, low fertile soil with a low fertility level and a high water infiltration rate exists [12]. Under Egypt’s current situation of water shortage and food insecurity, increasing the productivity of unit land and water in these soils is a challenge [12]. The attributes of soils have a considerable influence on the abundance of plant nutrients [13]. In many parts of the globe, sandy soils are employed in agriculture [14]. Among all deciduous fruit crops, the kernel-using apricot is thought to be one of the most drought-tolerant, thriving in locations with sandy soil, limited water supplies, and inadequate irrigation systems [15]. However, water deficit, as well as soil type, have been observed to impact apricot quality and production [16].

Apricot fruit cultivars are classified into two types based on its sweet or bitter seeds or kernels taste [3]. Seed bitterness is mainly attributed to cyanogenic glucosides [17] *viz.* toxic amygdalin which upon hydrolysis yields poison hydrogen cyanide whose content is reported to be much higher in bitter kernels compared to sweet ones [3]. Despite being toxic, amygdalin at small levels can stimulate respiration, improve digestion, and may be used for the treatment of cancer [3] identified as vitamin B17 misnomer [18]. Other vitamins reported in apricot include niacin, and thiamine.

Major sugars to account for apricot fruit sweetness are glucose, fructose, in addition to disaccharides i.e., sucrose and maltose, and trisaccharide i.e., raffinose, though at much lower levels [3]. Further, apricot fruit is rich in organic acids, e.g., malic, citric and quinic acids [19], phenolic acids e.g., chlorogenic and neochlorogenic acids, in addition to flavonoids, e.g., 2,7 (+)-catechin, (−)-epicatechin, and quercetin-3-rutinoside [20]. With regards to aroma composition to add to the fruit agreeable flavor, more than 80 aroma compounds are identified in the volatile fraction of apricots predominated with terpenes, esters, lactones, aldehydes and alcohols [19]. β-Ionone, linalool, γ-decalactone, 3,7-dimethyl-1,6-octadien, phenylacetaldehyde, hexanal, γ-dodecalactone, and γ-octalactone are identified as the compounds determining the aromatic profile of the apricot. β-Ionone and linalool could be responsible for the floral characteristic of apricot while lactones provide peach-like and coconut-like odors present in apricots [21]. Being distinguished by its high dietary value, apricot fruit is widely consumed either fresh or dried or in processing industry *viz.* juice, jam, puree, jelly, marmalade, fruit yogurt, and brandy [3,22,23].

The apricot kernel is a byproduct of apricot fruit weighing up to one third of the stone weight [24], and thus to be considered as valuable byproduct [3]. Sweet kernels are used either raw or roasted as an appetizer, salted tidbits, in baked products and as a substitute for nuts where its chemical composition is most similar to that of almond [6,24], besides its use in the confectionery industry for preparation of persipan; a sweetened paste made from roasted apricots, or peach kernels [6,25]. Likewise, the kernel is especially valued for the oleo-chemical industry due to its oil content. Apricot kernels are utilized both for the production of apricot kernel oil and oil cake [24]. Apricot kernel represents additionally a substantial source of dietary proteins predominated with albumin, essential amino acids, e.g., arginine and leucine, glutamic acid as major nonessential amino acid carbohydrates, lipids and minerals [3,22]. Apricot kernels are reported to be dominated by oleic and linoleic acid to amount for 92% of total fatty acids [6,24].

Concerning *Prunus armeniaca* L., this is the first metabolomics approach to address heterogeneity in different Egyptian cultivars in the context of its seed taste and type of soil used in cultivation. For analysis of food, several modern analytical approaches are increasingly implemented including that of large scale metabolomics aiming at the detailed characterization of metabolites within plant specimens in a rather untargeted, comprehensive manner [2]. For metabolomics profiling in fruits and seeds, gas chromatography coupled to mass-spectrometry (GC/MS) is commonly adopted to characterize its nutrients profile post silylation [2,26]. Primary metabolites in *P. armeniaca* L. fruit and seed mediate for their nutritive value, an objective of profiling its primary metabolome seems warranted to provide better insight into its metabolites composition. With regards to aroma composition, unlike traditional methods for volatiles extraction, such as distillation, the coupling of GC/MS to headspace solid phase micro-extraction provides a gentle and effective method for volatiles characterization (SPME). SPME outperforms previous approaches in terms of increased volatiles recovery using a fused-silica fibre, followed by the ultimate desorption of these analytes, allowing for the identification of less prevalent volatiles [2].

For better interpretation of such huge datasets, multivariate data analyses are often used, e.g., principal component analysis (PCA), in addition to supervised methods, *viz.* orthogonal projection to least squares discriminant analysis (OPLS-DA), which can simplify metabolite data complexity and facilitate sample classification [26], and identification of biomarkers. The objective of the current study is to employ chemometric tools for the first time to assess chemical determinants in Egyptian *P. armeniaca* L. fruit and seed to account for their nutritive and sensory characters in the context of different fruit cultivars type and soil conditions.

## 2. Materials and Methods

### 2.1. Plant Material, SPME and Chemicals

*Prunus armeniaca* L. fruits and seed kernels grown in different soil types *viz.* Manshiet Al-Ammar bitter apricots (AP-B_1_-F, AP-B_1_-S), Al-Amar Al-Kubra bitter apricots (AP-B_2_-F, AP-B_2_-S), Al-Amar Al-Kubra sweet apricots (AP-S_2_-F, AP-S_2_-S) (Muddy soil) and Nubaria mountain bitter apricots (AP-M_3_-F, AP-M_3_-S) (Sandy soil) were collected from Menoufia governorate in Egypt in June 2020, detailed sample information presented in Table 1. 

The collected samples are cultivars populations grown in Egypt, abbreviations refer to Apricot-bitter-fruit (AP-B-F), Apricot-bitter-seed kernel (AP-B-S), apricot-sweet-fruit (AP-S-F). All trees were around 10–12 years old; cultivation area is in Manshiyat al Amar, Qalyubia Egypt 9 m above sea level and located at 30.47° N 31.18° E with an average of 310 monthly hours of sunshine over the year, the temperature typically varies from 49° F to 97° F and is rarely below 45 °F or above 103 °F. Humidity ranges from 50 to 60%. Apricot trees grown in clay soil that is colored black, highly compact and of moderate fertility. Aeration level is moderate and trees are irrigated with flood irrigation method. Apricot trees attain an average height of 5–6 m. In contrast, apricot trees grown in sandy soil that is colored yellow, weakly compacted mostly composed of sand and of low fertility. Aeration level is high and trees are irrigated with drip irrigation method. Apricot trees attain an average height of about 2 m.

SPME fibers of stable flex coated with divinylbenzene/carboxen/polydimethylsiloxane (DVB/CAR/PDMS, 50/30 µm) or PDMS (polydimethylsiloxane) were purchased from Supelco (Oakville, ON, Canada). All other chemicals, volatile standards and sugars were purchased from Sigma Aldrich (St. Louis, MO, USA). Voucher specimens of fruits and seed kernels are kept in the Pharmacognosy department, Faculty of Pharmacy, Cairo University (Giza, Egypt).

### 2.2. Sample Preparation

Fruits were first manually pitted to remove seed kernels from fruit stony outerpart. Fruits and seed kernels were then lyophilized using Benchtop Freeze Dryer, LYO60B-1PT till complete dryness and powdered using Kenwood KHH326BK Multione Mixer 1000 W.

### 2.3. GC–MS Analysis of Silylated Primary Metabolites

100 mg of finely powdered sample (for both fruits and seed kernels) was extracted with 5 mL 100% methanol with sonication for 30 min using Branson CPX-952-518R set at 36 °C, (Branson Ultrasonics, Carouge, SA Switzerland.) and regular shaking, followed by centrifugation (LC-04C 80-2C regen lab prp centrifuge, Zhejiang, China) at 12,000× *g* for 10 min to eliminate debris. For evaluation of biological replicates, 3 independent samples for each apricot fruit and seed kernel were analyzed under the same conditions. Then, 100 µL of the methanol extract was kept in opened screw-cap vials and left to evaporate under stream of nitrogen gas until full dryness. For derivatization, 150 µL of N-methyl-N-(trimethylsilyl)-trifluoroacetamide (MSTFA) previously diluted 1/1 with anhydrous pyridine was mixed with the dried methanol extract and incubated (Yamato Scientific DGS400 Oven, QTE TECHNOLOGIES, Hanoi, Vietnam) for 45 min at 60 °C previous analysis using GC–MS. Separation of silylated derivatives was completed on a Rtx-5MS Restek, Bellefonte, PA, USA (30-m length, 0.25-mm inner diameter and 0.25-m film). Analysis of these primary metabolites followed the exact protocol detailed in [2,26]. Soluble sugars, amino acids, organic acids and fatty acids were quantified using standard curves of glucose, glycine, citric and stearic acids and results were expressed as mg/g. Four serial dilutions were prepared from 10 to 600 ug/mL for establishing the standard curves. Calibration curves for glucose, glycine, citric acid and stearic acids displayed 0.9948 correlation coefficient.

### 2.4. SPME–GC–MS Volatile Analysis

Dried, finely powdered fruits were placed in SPME screw-cap vials (1.5 mL) spiked with 10 µg (*Z*)-3-hexenyl acetate with fibers inserted manually above and placed in an oven (Yamato Scientific DGS400 Oven, QTE TECHNOLOGIES, Hanoi, Vietnam) kept at 50 °C for 30 min. HS-SPME analysis of the volatile compounds was performed as reported in [2,26]. The fiber was subsequently withdrawn into the needle and then injected manually into the injection port of a gas chromatography–mass spectrometer (GC–MS). GC–MS analysis was adopted on an Agilent 5977B GC/MSD (Santa Clara, CA, USA) equipped with a DB-5 column (30 m × 0.25 mm i.d. × 0.25 µm film thickness; Supelco, Bellefonte, PA, USA) and coupled to a quadrupole mass spectrometer. The interface and the injector temperatures were both set at 220 °C. Volatiles elution was carried out using the following gradient temperature program: oven was set at 40 °C for 3 min, then increased to 180 °C at a rate of 12 °C/min, kept at 180 °C for 5 min, finally increased at a rate of 40 °C/min to 240 °C and kept at this temperature for 5 min. Helium was utilized as a carrier gas with a total flow rate of 0.9 mL/min. For ensuring complete elution of volatiles, SPME fiber was prepared for the next analysis by placing it in the injection port at 220 °C for 2 min. For assessment of biological replicates, three different samples for each *P. armeniaca* fruits and seed kernels were analyzed under the same conditions. Blank runs were made during sample analyses. The mass spectrometer was adjusted to EI mode at 70 eV with a scan range set at *m*/*z* 40–500.

### 2.5. Metabolites Identification and Multivariate Data Analyses

Identification of volatile and non-volatile silylated components was performed by comparing their retention indices (RI) in relation to n-alkanes (C6-C20), mass matching to NIST, WILEY library database and with standards if available. Peaks were first deconvoluted using AMDIS software (www.amdis.net, accessed on 23 April 2022) before mass spectral matching. Peak abundance data were exported for multivariate data analysis by extraction using MET-IDEA software (Broeckling, Reddy, Duran, Zhao, and Sumner, 2006). Data were then normalized to the amount of spiked internal standard (Z)-3-hexenyl acetate, pareto scaled and then subjected to principal component analysis (PCA), hierarchical clustering analysis (HCA) and partial least squares discriminant analysis (OPLS-DA) using SIMCA-P version 13.0 software package (Umetrics, Umeå, Sweden). All variables were mean-centered and scaled to Pareto variance.

### 2.6. Statistical Analysis

All data were expressed as mean ± S.D. of three replicates in each group. Statistical analysis was performed using one-way analysis of variance (ANOVA) according to the model; Yijk = µ + Ti + Eij (Yijk = any observation. µ = overall mean. Ti = the fixed effect of the estimate. Eij = general error) using general model program SPSS Statistics, Version 26 SPSS, Inc., Chicago, Ill), 2019. Significance values amongst groups were calculated using Tukey’s test at *p* < 0.05.

## 3. Results and Discussion

### 3.1. Primary Metabolites Profiling of Apricot Fruits and Seed Kernels via GC-MS Analysis (Post-Silylation)

**Figure 1:**
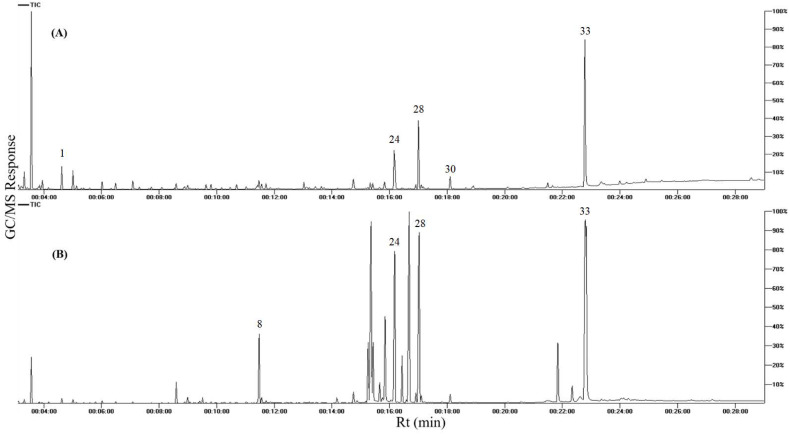
GC/MS chromatogram of silylated metabolites in apricot seed kernels (**A**) and fruits (**B**). The corresponding metabolite names for each peak follow that listed in Table 2.

GC–MS analysis was applied post-silylation to afford an inclusive picture of primary metabolites accounting for fruits and seeds’ nutritional value and or organoleptic characters (Table 1). A total of 36 peaks (Table 2, Figure 1) where detected in fruits and seed kernels, belonging to sugars (mono- and disaccharides), fatty acids, organic and amino acids. 

Representative GC-MS chromatograms of apricot fruits and seed kernels primary metabolites are presented in Figure 1.

#### 3.1.1. Sugars

Sugars were the most dominant primary metabolite class identified in all apricot fruits and seed kernels comprising 15 peaks mostly represented by mono- and di-saccharides ranging from 189.98–268.32 mg/g in fruits and from 11.9–58.1 mg/g in seed kernels. D-Glucose (peak 24 and 28) was the main sugar detected in all samples, except for apricot sweet seed kernel AP-S_2_-S (muddy soil) and apricot mild bitter fruit AP-M_3_-F (sandy soil), with sucrose (peak 33) being the predominant sugar detected at 10.7 mg/g and 91.1 mg/g respectively. D-Glucose was detected in all apricot specimens ranging from 67.1–132.3 mg/g in fruits, and with larger variance in seed kernels ranging from 0.51–35.7 mg/g.

By comparing each organ separately, it was observed that the lowest glucose level (67.1 mg/g) was detected in apricot mild bitter fruit AP-M_3_-F (sandy soil) in comparison to other fruits (AP-B_1_-F, AP-B_2_-F and AP-S_2_-F) grown in muddy soil. In contrast in seed kernels, apricot sweet seed kernel AP-S_2_-S showed the lowest glucose level (0.51 mg/g) compared to sucrose (10.78 MG/G). However, the highest glucose level (132.3 mg/g) was detected in apricot bitter fruit AP-B_1_-F cultivated in muddy soil.

Next to glucose, fructose (peaks 20 and 21) and galactofuranose (peak 22) were detected in all specimens though at lower levels, found richer in fruits than seed kernels. Sugars detected at trace quantities included xylose and allofuranose in both fruits and seed kernels.

Sucrose and fructose are the prominent contributors to fruits sweetness in case of apricot, being the most important sensory quality for consumers [27]. Apricot fruits from Egypt sugar results are in accordance with that reported by Bae et al., [28] in which higher glucose level than sucrose has been reported in apricot fruits from other regions [28]. Extensive studies conducted in different apricot growing regions indicate that cultivar type determines the fruit sugar level rather than origin [27,29]. Moreover, variability in sugar levels among different allocations can be attributed to differences in the soil, nutrients as well as climatic conditions, in addition to fruit development and ripening [30,31,32]. Other sugars such as sorbitol, mannose, maltose and raffinose reported in apricot fruits in varying levels were not found in our samples [33]. Regarding sugar alcohols, inositol (peak 30) was detected in apricot fruits (0.9–1.7 mg/g) and seed kernels (0.2–0.9 mg/g). Other sugar alcohols included myo-inositol (peak 29) and mannitol (peak 25) detected at low levels in fruits and seed kernels and suggestive that apricot does not present a good source of sugar alcohols compared to other *Prunus* fruits such as *P. domestica* [34].

#### 3.1.2. Organic Acids

Organic acids play a major role in defining fruit taste in addition to their action as an acidulant preservative to inhibit the growth of microorganisms and improve fruits shelf life [35]. Comparable organic acids level was detected in all examined apricot fruits, with malic acid (peak 8) as major component, that exhibited higher levels in fruits (21.9–29.8 mg/g) than seed kernels (0.15–2.4 mg/g). No significant difference was observed in malic acid level among the different fruit types. Malic acid is a common acid in fruits and vegetables [36] that exhibit significant antioxidant activity [37], asides from acting as a bioavailability enhancer of minerals such as iron [36]. next to malic acid, tiglic acid (peak 1) was also detected in all apricot fruits and seed kernels with no significant variance among them. Whether apricot seed can present a source of tiglic acid as in croton oil with potential industrial applications as flavoring agent [38] needs further investigations.

Other acids detected at lower levels include fumaric (peak 6) and quininic acids (peak 10) in all apricot fruits and seed kernels. Organic acids profiling in Egyptian apricot are in accordance to those reported in literature regarding the presence of malic acid as major form [27]. However, no studies reported the existence of tiglic acid in apricot fruits and seeds. Various studies reported the presence of other acids detected in this study including isocitric acid, succinic acid, fumaric acid, shikimic acid [20]. Levels of organic acids and sugars play an important impact on fruit flavor and quality [39]. Apricot fruit is highly appreciated by consumers, as fruit exhibits a perfect balance between sugars and organic acids, combined with a strong and rich flavor and aroma [40].

#### 3.1.3. Amino Acids

Free amino acids were detected at low amounts in all apricot fruits (5.3–6.8 mg/g) and seed kernels (2.4–8.7 mg/g) represented by L-alanine (peak 11) that displayed higher concentrations in fruits when being compared to seed kernels.

#### 3.1.4. Free Fatty Acids

Free fatty acids were detected at low quantities in all examined fruits ranging from 1.8–5.0 mg/g, and from 1.05–1.7 mg/g in seed kernels. Major forms included palmitic acid (peak 34), linoleic acid (peak 35) and vaccenic acid (peak 36), with highest level of linoleic acid found in apricot bitter fruits AP-B_1_-F (3.3 mg/g) and AP-B_2_-F (3.1 mg/g) growing in muddy soil. Palmitic acid accounted for ca. 8–10% of dietary energy [41], whereas vaccenic acid is a positional and geometric isomer of oleic acid [42], a predominant trans fatty acid found in animal fat [43]. These results are in accordance with fatty acids composition previously reported in apricot fruits and seeds from other origins [44].

### 3.2. Multivariate Data Analyses of Primary Silylated Metabolites of the Different Apricot Cultivars Fruit and Seed Kernel Cultivated in Different Soil Types

Chemometric tools were employed for the holistic assessment of primary metabolites heterogeneity amongst apricot cultivars, particularly given the significant number of peaks as variables in 8 selected specimens, each of which is represented by three biological replicates, and thus totaling 24 samples.

**Figure 2:**
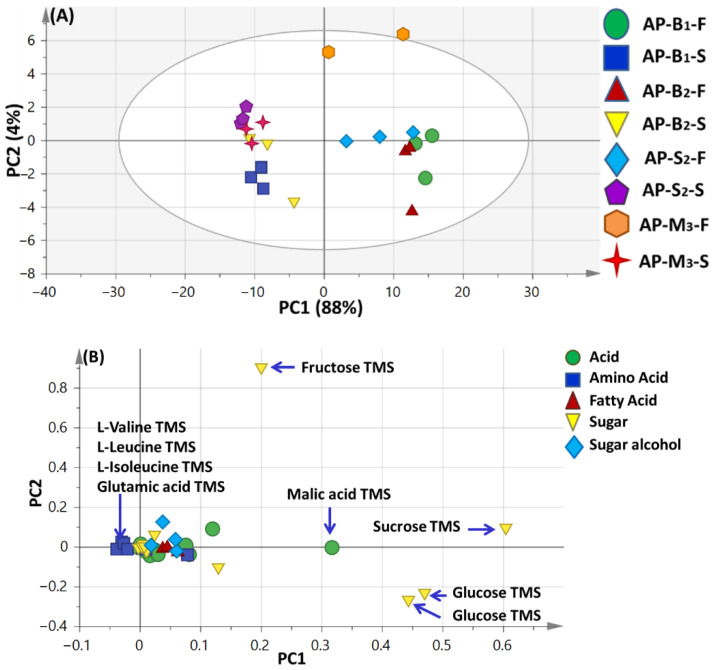
Principal component analyses of silylated metabolites in seed kernel and fruit extracts as analyzed by GC-MS (*n* = 3). (**A**) Score plot of PC1 vs. PC2 scores. (**B**) Loading plot for PC1 and PC2 contributing mass peaks and their assignments. AP-B1-F; Manshiet Al-Ammar bitter apricot fruits (Muddy soil), AP-B1-S; Manshiet Al-Ammar bitter apricot seeds (Muddy soil), AP-B2-F; Al-Amar Al-Kubra bitter apricot fruit (Muddy soil), AP-B2-S; Al-Amar Al-Kubra bitter apricot seed (Muddy soil), AP-M3-F; Nubaria mountain bitter apricot fruit (Sandy soil), AP-M3-S; Nubaria mountain bitter apricot seed (Sandy soil), AP-S2-F; Al-Amar Al-Kubra sweet apricot fruit (Muddy soil), AP-S2-S; Al-Amar Al-Kubra sweet apricot seed (Muddy soil).

Principal component analysis (PCA) was illustrated by two orthogonal PCs, accounting for 92% of the total variance prescribed by PC1 and PC2 (Figure 2A).

An obvious segregation between apricot seed kernels from fruits could be observed along PC1, with apricot seed kernel specimens positioned with negative score values (left side in PC1), whereas apricot fruit specimens clustered with positive score values (right side in PC1). Examination of the loading plot (Figure 2B) revealed that sugars *viz.* fructose, sucrose and glucose as well as acids *viz*. malic acid contributed the most for apricot fruit specimens’ segregation with glucose, sucrose and fructose serving as major contributors to fruit sweetness, whereas amino acids *viz.* valine, leucine, isoleucine and glutamic acids found more abundant in apricot seed kernels suggestive for the sour taste of seed kernels compared to fruits.

#### 3.2.1. Multivariate Data Analyses of the Primary Silylated Metabolites in Fruits and Seed Kernels Models

For better segregation and to help identify variation within each organ, apricot seed kernel and fruit specimens were modeled separately one at a time (Supplementary Figures S1A and S2A, respectively. The seed kernels PCA model was illustrated by two orthogonal PCs, justifying 82% of the total variance, with clear discrimination of AP-S_2_-S (sweet seeds of apricot) and AP-M_3_-S (mild bitter seeds of apricot) at the left side of PC1 separable from other apricot bitter seed kernel specimens positioned in the middle and on right side of PC1. Examination of the loading plot (Supplementary Figure S1B revealed that variables corresponding to sugars *viz.* fructose, glucose and sucrose were found to be most enriched in AP-B_1_-S (seeds of Manshiet Al-Ammar apricots (Muddy soil) and AP-B_2_-S (seeds of Al-Amar Al-Kubra apricots (Muddy soil) specimens. Modelling of nutrient metabolites suggest that cultivar type overcomes other variables in apricot seed kernels’ segregation with sweet seed kernels being most distant from others.

Likewise, to help identify variation within apricot fruit specimens, another PCA model (Supplementary Figure S2A was employed. The main principal component (PC) to differentiate specimens in PCA, that is PC1 to account for 62% of the total variance, with apricot fruit (mild bitter seeds cultivar) cultivated in sandy soil (AP-M_3_-F) found positioned at one side (positive PC1 score value), while the other 3 fruit samples in muddy soil segregated on the left side (negative PC1 score value) and in the middle. Examination of the loading plot (Supplementary Figure S2B revealed that variables corresponding to sugars *viz.* fructose was found to be most enriched in AP-M_3_-F specimen. Whereas, malic acid, glucose and sucrose were more abundant in other fruit specimens. ANOVA (Supplementary Table S1) revealed significant differences in the percentile levels of organic acids i.e., malic acid, amino acids i.e., L-Alanine, L-Valine, L-Leucine, L-Isoleucine and Glutamic acid, sugars *viz.* glucose, sucrose and galactofructose among the different apricot specimens with a confidence level of 95%.

AP-M_3_-F cultivar contained organic acids i.e., malic acid and sugars i.e., sucrose at a significantly higher level compared to other cultivars at *p* < 0.05. AP-B_1_-F cultivar contained a significantly higher level of sugars *viz.* glucose and sucrose at significantly higher levels than other cultivars at *p* < 0.05. AP-M3-S cultivar contained amino acids *viz.* valine, leucine, isoleucine & glutamic acid at significantly higher levels than other cultivars at *p* < 0.05.

Modelling of primary metabolites suggest that soil type overcomes other variables in apricot fruit segregation with fruits grown in sandy soil being most distant from others.

#### 3.2.2. OPLS-DA Analysis of Apricot Fruit Cultivar Propagating in Sandy Versus Muddy Soil Primary Silylated Metabolites Dataset

**Figure 3:**
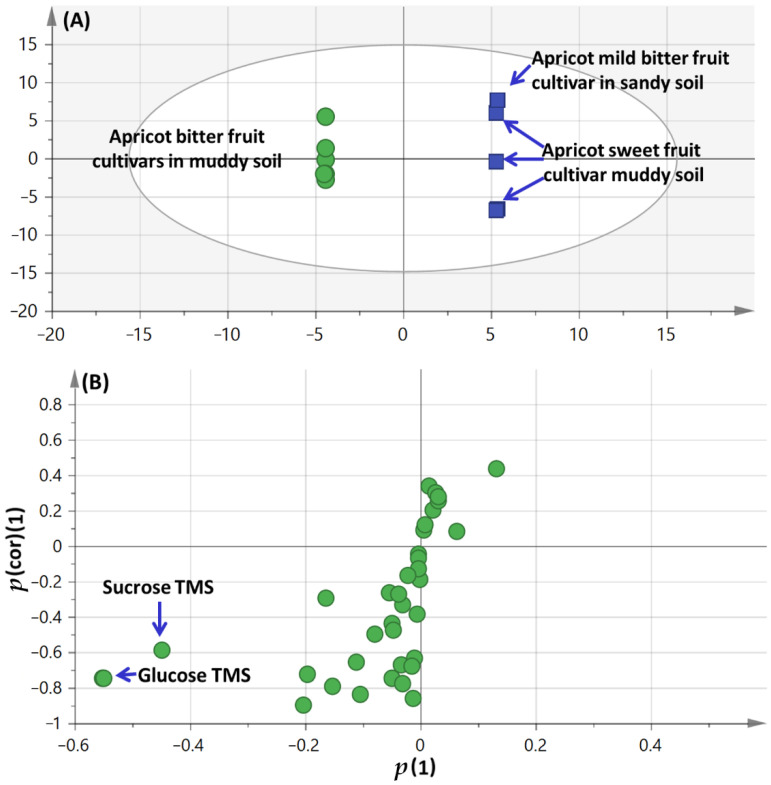
GC–MS-based OPLS-DA score plot (**A**) derived from modeling silylated primary metabolites of apricot fruit cultivar grown in sandy versus muddy soil (*n* = 3). The respective loading S-plots (**B**) showing the covariance *p* (1) against the correlation *p*(cor) (1) of the variables of the discriminating component of the OPLS-DA model. Cut-off values of *p* = 0.32 was used. Designated variables are highlighted and identifications are discussed in the text.

The supervised orthogonal partial least square discriminant analysis (OPLS-DA) was further employed to assess apricot fruit classification based on soil type (sandy and muddy), which failed to separate in PCA analysis. Hence, a model of apricot fruit cultivars cultivated in sandy soil (AP-M_3_-F) against apricot fruit cultivars cultivated in muddy soil (AP-B_1_-F, AP-B_2_-F and AP-S_2_-F) in another class was constructed (Figure 3A). OPLS model exhibited Q^2^ = 0.64 indicating the model predictability, and total variance coverage of 82% (R^2^ = 0.82). The respective loading S-plot (Figure 3B) revealed that glucose and sucrose (peaks 26 and 39) were enriched in apricot bitter fruit cultivars compared to other ones, though with non-significant *p* value of 0.32.

### 3.3. Apricot Fruit Headspace Volatile Analysis via SPME–GC–MS

**Figure 4:**
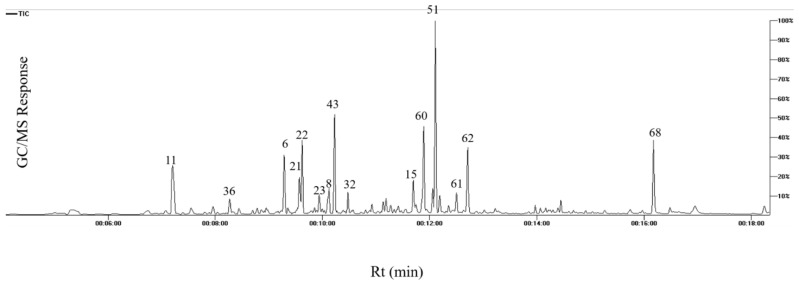
GC/MS chromatogram volatile metabolites in apricot fruits. The corresponding volatile names for each peak follow that listed in Table 3.

Considering apricot fruit unique aroma and to characterize it profile for the first time from Egyptian origin, headspace–solid-phase micro-extraction (HS-SPME) was employed as a relatively cold extraction method compared to steam distillation [45]. SPME analysis led to the identification of 70 volatiles belonging to alcohols, aldehydes, esters, ethers, ketones, oxides, inones, lactones, fatty acids, sesqui- and monoterpenes (Table 3, Figure 4). Fruit aroma is the result of special assortment and mixture of different volatile components that determine the overall aroma properties [21]. Quality and preferences of apricot fruits are appreciated by consumers for their unique aroma which warrants for its detailed characterization [46].

#### 3.3.1. Lactones

Lactones amounted for the major class of volatiles in apricot fruits at ca. 24, 38, 37 and 14% in AP-B_1_, AP-B_2_, AP-M_3_ and AP-S_2_, respectively. γ-Decanolactone (peak 60) was the predominant form in all fruits representing 31.2% of the total lactones in AP-M_3_, followed by 26.3% and 17.1% in AP-B_2_ and AP-B_1_, respectively versus lowest in AP-S_2_ (5%). The highest amount of γ-decanolactone was detected in mild bitter apricot fruit growing in sandy soil followed by bitter ones in muddy soil. Other identified lactones included δ-decalactone, dihydroactinidiolide and γ-dodecalactone though detected at lower levels. Lactones provide peach-like and coconut like odors present in apricots mostly attributed to γ-dodecalactone and δ-decalactone [32,47].

#### 3.3.2. Fatty Acids/Esters

Fatty acid esters presented the second most abundant volatile class in apricot fruits, with highest levels (25.4%) in apricot bitter fruit AP-B_2_, followed by apricot sweet fruit AP-S_2_ and apricot bitter fruit AP-B_1_ at ca. 13% grown in muddy soil. In contrast, lowest level was detected in apricot mild bitter fruit growing in sandy soil AP-M_3_. Methyl palmitate (peak 68) was the major identified fatty acyl ester in all samples; with the highest level detected in apricot bitter fruit AP-B_2_ accounting for 20.3% of the total identified fatty acyl esters.

Other identified methylated fatty acids detected in apricot aroma in all fruit samples included methyl laurate, methyl myristate, myristic acid, methyl palmitoleate. Pintea et al. reported the predominance of linoleic acid, (47%), palmitic acid (32.7%), and linolenic (17%) in various apricot cultivar in Romania in contrast to our results, where palmitic acid is detected at low levels with the absence of linoleic and linolenic acids [48].

#### 3.3.3. Alcohols

Alcohols are also among the major aroma compounds identified in various apricot samples, detected at comparable levels ca. 11% in all fruits except in apricot mild bitter fruit AP-M3 growing in sandy soil where it reached 24%. In particular, α-terpineol (peak 6) represented the major form with highest level (13.2%) in AP-M3. In contrast to all apricot samples, AP-M3 showed the highest level of linalool (8.8%) compared to other samples and suggestive that AP-M3 grown in sandy soil has the most distinct aroma compared to other specimens considering linalool strong aroma of a floral lavender-like, fresh, citric odor [48]. α-Terpineol that exhibited a heavy floral, lilac-type odor and linalool (floral odour) are indeed reported among the major contributors to apricot aroma growing in Italy and France [47,49]. Compared to phenylethyl alcohol, p-cymen-8-ol and geraniol were detected in all apricot samples. (*Z*)-hex-3-en-1-ol was only detected in apricot bitter fruit AP-B_2_ and apricot sweet fruit AP-S_2_ propagated in muddy soil, whereas p-menth-1-en-9-ol was identified exclusively in AP-M_3_ and AP-S_2_. Previous studies reported that geraniol and (*Z*)-hex-3-en-1-ol (green leaf odour) are among the volatile constituents that play a role in apricot aroma [32,47].

#### 3.3.4. Aldehydes

β-Cyclocitral (peak 22) and p-anisaldehyde (peak 23) were the most abundant aldehydes, being more enriched in sweet apricot fruit AP-S_2_ at 2.4% and 7.1% respectively, of the total aldehydes. β-Cyclocitral results from thermal, photo oxygenation or enzymatic degradation of β-carotene the main carotenoid pigment in apricot, and to further account for the fruity and especially citrus note of apricot aroma [49]. Other detected aldehydes included 5-formylfurfural, benzaldehyde, 2,5-dimethyl-, n-decanal, safranal, 5-hydroxymethylfurfural, α-citral, (*E*)-cinnamaldehyde, piperonal, methyleugenol and 4-methoxycinnamaldehyde.

#### 3.3.5. Ionones

Ionones are among the main unique volatile classes in apricot to account for 7–14% of the total aroma composition. β-Ionone (peak 51) was the most abundant detected at (13.7%) in apricot bitter fruit AP-B1 followed by apricot sweet fruit AP-S2 (11.5%) and apricot bitter fruit AP-B2 (6%). β-ionone is one of the key characteristic determinants contributing with flowery and raspberry notes [21] in apricot. Other ionones detected at lower levels included α-ionene, dehydro-ar-ionene, megastigma-4,6(*z*),8(*e*)-triene and dihydro-β-ionone. The presence of different ionones in apricot is derived from the degradation of carotenoids rich in apricot fruits that increase during fruit development [32].

#### 3.3.6. Ethers/Oxides

Ethers were detected in different apricot fruits with highest levels 20.5% in apricot sweet fruit AP-S_2_ represented mainly by (*E*)-anethole (peak 43) that accounted for 20% of the total ethers in AP-S_2_ versus lowest level in apricot bitter fruit AP-B_2_ 2.9%. (*E*)-Anethole is the major anise flavor compound with sweet and distinct taste, with significant antimicrobial, antioxidant and antispasmodic activities [50]. Nerol oxide was the only identified oxide in apricot fruits though at trace levels ranging from 0.01 till 0.7%.

#### 3.3.7. Ketones

Ketones amounted for 4.7%, 4.4%, 2.9% and 10.2% of the total volatiles in apricot fruits AP-B_1_, AP-B_2_, AP-M3 and AP-S_2_ respectively. Geranyl acetone (peak 15) was the most abundant ketone, is a carotenoid degradation product with sweet floral rose like odor previously isolated from apricot fruits [21]. Next to geranyl acetone, anisketone was also detected in AP-B_1_ and AP-S_2_ (2.7 and 3.6%, respectively) versus much lower levels (ca. 0.3% in AP-B_2_, AP-M_3_). In contrast, AP-B_2_ and AP-S_2_ showed higher isophorone level, a ketone previously identified in apricot fruit [48] and have been found uniquely in Egyptian date fruits [51]. Various ketones that are not previously reported as anisketone, along with (*Z*)-Jasmone and turmerone were detected in all fruit samples though at low levels.

#### 3.3.8. Esters

Ester levels detected in apricot fruits aroma were found comparable among fruits at ca. 2% and suggestive that they do not contribute to fruits aroma although being of agreeable smell. Methyl benzoate (peak 36) was the most abundant ester, followed by benzyl acetate, methyl salicylate, α-terpinyl acetate and benzyl benzoate detected at trace levels.

#### 3.3.9. Aliphatic/Aromatic Hydrocarbons

Aliphatic hydrocarbons amounted for 0.5–1% of the volatile composition in fruits, except apricot bitter fruit AP-B_1_ that showed much higher levels (8.9%) represented by octadecane (peak 58) as major form (8.3%). Aliphatic hydrocarbons were previously identified in apricot fruit volatiles [52]. Among aromatics, β-methylnaphthalene (peak 32), was detected as major form in AP-B_1_ (1.9%). p-Cymenene was present at trace levels in accordance with other reported data in apricot [48] in addition to α-methylnaphthalene, diphenyl ether and dimethyl naphthalene detected at trace levels.

### 3.4. Multivariate Data Analyses of Sweet and Bitter Apricot Fruit Cultivars Aroma Profile Cultivated in Different Soil Types

Heterogeneity in the volatile distribution (70 peaks for volatiles) of apricot fruit accessions was explored in a more holistic way using PCA, as employed in the primary metabolites dataset (Supplementary Figure S3). PCA score plot of the apricot fruit cultivars aroma profile (Supplementary Figure S3A) was ascribed by two main vectors, i.e., PC1 and PC2, both accounting for 57% of the total variance. The performance of the classification model was validated by the computed parameters “R2 (0.34)” and “Q2 (−0.1)”, which indicated the low prediction power of the model, and suggestive that aroma compounds cannot be used for fruits classification. The main volatile responsible for fruit specimens’ segregation were revealed from the corresponding PCA loading plot (Supplementary Figure S4B) to include E-anethole (peak 25), being most enriched in sweet apricot fruit cultivar (AP-S_2_-F).

HCA analysis was employed in order to better assess volatiles heterogeneity among apricot fruit specimens. HCA dendrogram showed two distinct clusters (Supplementary Figure S4C), where sweet apricot fruit cultivar (AP- S_2_-F), clustered in group 1, while bitter apricot fruit cultivars (AP-B_1_-F, AP-B_2_-Fand AP-M_3_-F) clustered in group 2. However, the clustering of all bitter apricot fruit cultivars together in group 2 indicated that HCA failed to characterize the impact of soil type based on their volatile metabolites.

#### OPLS-DA Analyses of Volatile Metabolites in Different Apricot Fruit Cultivars

**Figure 5:**
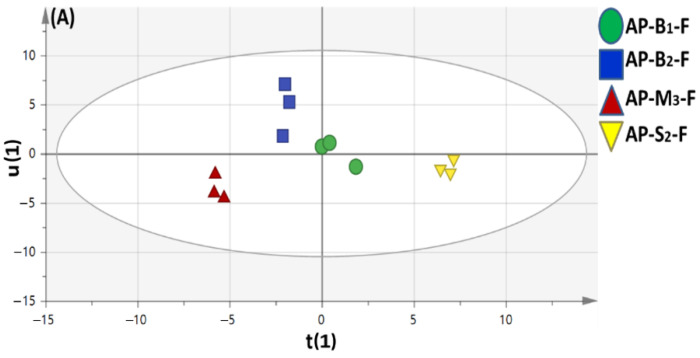

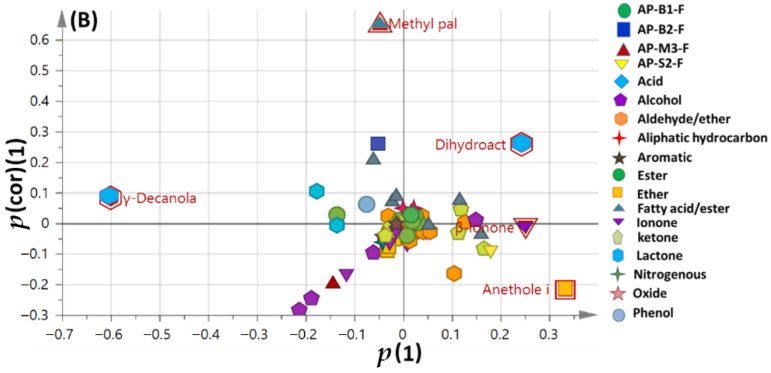
GC–MS-based OPLS-DA score plot (**A**) derived from modeling volatile metabolites of different apricot fruit cultivars (*n* = 3). The respective loading plots (**B**) showing the covariance *p* (1) against the correlation *p*(cor) (1) of the variables of the discriminating component of the OPLS-DA model.

OPLS-DA modeling of apricot fruit cultivars aroma profile (Figure 5A) was attempted in order to assess the variation amongst different apricot fruit cultivars in context to both cultivar type (sweet or bitter seed) and soil type (muddy or sandy).

The performance of the developed classification model was validated by the computed parameters “R2 (0.61)” and “Q2 (0.22)”, which indicated improved prediction power than PCA model (Supplementary Figure S4A). The observed segregation in the derived score plot (Figure 5B) was ascribed to the enrichment of sweet apricot fruit seed cultivar (AP- S_2_-F) in (E)-anethole (peak 42), β-ionone (peak 51) and dihydroactinidiolide (peak 62) versus abundance of the lactone, γ-decanolactone (peak 60) and methyl palmitate (peak 68) in bitter apricot fruit cultivars. γ-Decanolactone is a characteristic aroma compound in Rosaceae seeds and extend herein to include apricot [49].

Further and for better segregation of sweet apricot fruit cultivar (AP- S_2_-F) from bitter apricot fruit cultivars (AP-M_3_-F, AP-B_1_-F and AP-B_2_-F), supervised (OPLS-DA) score plot Supplementary Figure S4A was implemented. Model showed Q^2^ = 0.57 indicating model predictability of the OPLS score plot and 90% of the total variance coverage (R^2^ = 0.90). The respective loading S-plot (Supplementary Figure S4B) revealed that anethole isomer (peak 43) was enriched in apricot sweet fruit cultivar compared to bitter ones, later being enriched in lactones *viz.* δ-decalactone (peak 61) and γ-decanolactone (peak 60), with significant p value of 0.02.

ANOVA test was performed for discrimination assessment amongst volatile metabolites (Supplementary Table S2). Significant differences were revealed in the percentile levels of ionones *viz.* β-ionone & lactones *viz.* dihydroactinidiolide among the different apricot fruit cultivars with a confidence level of 95%.

AP-B_1_-F cultivar contained a significantly higher level of ionones *viz.* β-ionone, fatty acid/esters *viz.* methyl palmitate & lactones *viz.* dihydroactinidiolide compared to other fruit cultivars at *p* < 0.05.

## 4. Conclusions

The compositional heterogeneity in the primary and aroma composition of *P*. *armeniaca* fruits and seeds cultivars grown in Egypt in the context of its different cultivars and soil type (muddy and sandy) was investigated through a holistic untargeted GC–MS metabolomics approach coupled with multivariate data analyses for the first time. Results of GC–MS analysis post silylation led to the detection of 46 peaks, belonging to sugars, fatty acids, esters and organic acids. Both glucose and sucrose were enriched in apricot fruits when being compared to the seeds. Multivariate data analysis of the primary metabolome revealed that sugars contributed the most for apricot fruit specimen’s segregation, with sucrose and fructose serving as major contributors to fruit sweetness, whereas amino acids *viz*. valine, leucine, isoleucine and glutamic acids found more abundant in apricot seeds suggestive for the sour taste of seeds compared to fruits. Further, statistical analysis has been accomplished providing the statistically significant different variables among specimens *viz.* malic acid, glucose, sucrose, alanine, valine, leucine, isoleucine and glutamic acids. Modelling of primary metabolites suggested that cultivar type overcomes other variables in apricot seeds segregation with sweet seeds being most distant from others. A total of 70 volatiles were identified, with lactones, alcohols and esters representing the major classes of apricot volatiles accounting for its discrete aroma. The major identified aroma compounds contributing to the segregation between various apricot fruit cultivars were linalool, anethole, p-anisaldehyde, dihydroβ-Ionone, β-Ionone γ-decanolactone, dihydroactinidiolide, γ-dodecanolactone and methyl palmitate. Further, statistical analysis has been accomplished providing the statistically significant different variables among specimens *viz.* β-Ionone, methyl palmitate and dihydroactinidiolide. We acknowledge that our current study is limited to few cultivars, with future work to analyze other and or from other origins for comparison. Modelling of nutrient metabolites suggest that soil type overcomes other variables in apricot fruit segregation with fruits grown in sandy soil being most distant from others. Same metabolomics platform can indeed be used to assess other conditions such as climate, geographical origins and agricultural practices on apricot fruit and seed metabolome.

## Figures and Tables

**Table 1:** Origin of the different apricot fruits and seed kernels used in this study.

Fruit Codes	Seed Codes	Collection Location/Soil Nature	Type
AP-B1-F	AP-B1-S	Manshiet Al-Ammar apricots (Muddy soil)	Bitter
AP-B2-F	AP-B2-S	Al-Amar Al-Kubra apricots (Muddy soil)	Bitter
AP-M3-F	AP-M3-S	Nubaria mountain apricots (Sandy soil)	Bitter
AP-S2-F	AP-S2-S	Al-Amar Al-Kubra apricots (Muddy soil)	Sweet

AP-B1-F; Manshiet Al-Ammar bitter apricot fruits (Muddy soil), AP-B1-S; Manshiet Al-Ammar bitter apricot seeds (Muddy soil), AP-B2-F; Al-Amar Al-Kubra bitter apricot fruit (Muddy soil), AP-B2-S; Al-Amar Al-Kubra bitter apricot seed (Muddy soil), AP-M3-F; Nubaria mountain bitter apricot fruit (Sandy soil), AP-M3-S; Nubaria mountain bitter apricot seed (Sandy soil), AP-S2-F; Al-Amar Al-Kubra sweet apricot fruit (Muddy soil), AP-S2-S; Al-Amar Al-Kubra sweet apricot seed (Muddy soil).

**Table 2:** Silylated metabolites expressed in mg/g in apricot fruits and seed kernels analyzed via GC–MS, *n* = 3. For codes, refer to Table 1. ^a–d^ Different letters in the same line concerning *p. armeniaca* L. specimens indicate significantly different values (*p* < 0.05; Tukey’s test).

	Rt (min)	KI	Name	AP-B1-F	AP-B1-S	AP-B2-F	AP-B2-S	AP-S2-F	AP-S2-S	AP-M3 F	AP-M3-S
					Organic Acids (mg/g)						
1	4.614	1029	Tiglic acid (TMS)	3.76 ± 0.91	2.65 ± 0.26	2.80 ± 0.39	3.60 ± 1.10	2.84 ± 0.47	2.82 ± 0.63	2.71 ± 0.61	3.17 ± 0.95
2	5.378	1077	Lactic Acid (2TMS)	0.41 ± 0.05 ^b^	0.24 ± 0.15 ^abc^	0.45 ± 0.14 ^c^	0.15 ± 0.05 ^a^	0.25 ± 0.08 ^abc^	0.15 ± 0.04 ^c^	0.21 ± 0.08 ^abc^	0.17 ± 0.03 ^bc^
3	5.584	1089	Glycolic acid (2TMS)	0.34 ± 0.07	0.21 ± 0.00	0.23 ± 0.02	0.26 ± 0.09	0.22 ± 0.02	0.21 ± 0.06	0.20 ± 0.05	0.23 ± 0.06
4	5.784	1102	Pyruvic acid (2TMS)	2.61 ± 0.99 ^a^	0.05 ± 0.02 ^c^	1.48 ± 0.20 ^c^	0.06 ± 0.02 ^b^	1.46 ± 0.46 ^b^	0.07 ± 0.03 ^c^	0.88 ± 0.17 ^bc^	0.08 ± 0.00 ^c^
5	9.08	1322	Succinic acid (TMS)	0.01 ± 0.01 ^bc^	0.02 ± 0.00 ^a^	0.01 ± 0.00 ^bc^	0.01 ± 0.01 ^bc^	0.00 ± 0.00 ^c^	0.01 ± 0.01 ^bc^	0.00 ± 0.00 ^c^	0.00 ± 0.00 ^c^
6	9.498	1353	Fumaric acid (TMS)	3.14 ± 0.77 ^bc^	0.02 ± 0.01 ^b^	4.00 ± 0.92 ^b^	0.02 ± 0.01 ^bc^	4.95 ± 3.19 ^bc^	0.11 ± 0.04 ^b^	6.78 ± 4.04 ^a^	0.10 ± 0.02 ^b^
7	9.621	1362	Methylmaleic acid (2TMS) ester	0.36 ± 0.04 ^ab^	0.21 ± 0.05 ^b^	0.35 ± 0.07 ^ab^	0.29 ± 0.10 ^ab^	0.35 ± 0.03 ^ab^	0.37 ± 0.08 ^ab^	0.43 ± 0.22 ^ab^	0.55 ± 0.15 ^a^
8	11.453	1505	Malic acid (TMS)	29.81 ± 9.94 ^a^	0.15 ± 0.07 ^b^	24.89 ± 5.99 ^b^	0.56 ± 0.18 ^a^	21.90 ± 9.27 ^a^	2.40 ± 0.97 ^b^	22.64 ± 12.51 ^a^	1.62 ± 0.41 ^b^
9	15.361	1850	Isocitric acid (TMS)	1.45 ± 0.09 ^a^	0.09 ± 0.02 ^b^	1.50 ± 0.11 ^b^	0.08 ± 0.07 ^a^	1.51 ± 0.27 ^a^	0.01 ± 0.01 ^b^	1.12 ± 0.23 ^a^	0.02 ± 0.00 ^b^
10	15.816	1894	Quininic acid (5TMS)	2.52 ±1.14	1.77 ± 2.54	2.36 ± 1.10	1.14 ± 0.92	0.63 ± 0.08	1.24 ± 0.58	1.40 ± 0.27	0.70 ± 0.16
	Total organic acids			44.42	5.41	38.08	6.16	34.13	7.40	36.37	6.64
					Amino acids (mg/g)						
11	6.011	1116	L-Alanine (2TMS)	3.40 ± 1.86 ^a^	0.61 ± 0.11 ^b^	3.22 ± 0.19 ^ab^	1.13 ± 0.50 ^ab^	2.82 ± 0.79 ^ab^	1.87 ± 0.72 ^ab^	2.47 ± 1.48 ^ab^	1.47 ± 0.38 ^ab^
12	7.72	1226	L-Valine, N-(TMS)-, trimethylsilyl ester	0.39 ± 0.06 ^bcd^	0.24 ± 0.11 ^bcd^	0.20 ± 0.06 ^bcd^	0.34 ± 0.22 ^cd^	0.06 ± 0.03 ^d^	0.89 ± 0.39 ^ab^	0.85 ± 0.30 ^abc^	1.51 ± 0.40 ^a^
13	8.529	1284	L-Leucine, N-(TMS)-, trimethylsilyl ester	0.06 ± 0.02 ^c^	0.33 ± 0.13 ^bc^	0.05 ± 0.02 ^bc^	0.37 ± 0.28 ^c^	0.03 ± 0.01 ^c^	0.62 ± 0.33 ^ab^	0.16 ± 0.04 ^bc^	0.86 ± 0.21 ^a^
14	8.843	1306	L-Isoleucine, N-(TMS)-, trimethylsilyl ester	0.20 ± 0.03 ^bcd^	0.17 ± 0.08 ^bcd^	0.12 ± 0.02 ^bcd^	0.25 ± 0.15 ^cd^	0.06 ± 0.02 ^d^	0.59 ± 0.27 ^ab^	0.54 ± 0.21 ^abc^	0.87 ± 0.26 ^a^
15	8.878	1309	L-Proline (2TMS)	0.01 ± 0.01	0.00 ± 0.00	0.01 ± 0.00	0.00 ± 0.00	0.00 ± 0.00	0.00 ± 0.00	0.00 ± 0.01	0.00 ± 0.00
16	9.792	1374	L-Serine (2TMS)-, trimethylsilyl ester	0.99 ± 0.24 ^ab^	0.07 ± 0.03 ^d^	0.75 ± 0.32 ^cd^	0.17 ± 0.09 ^abc^	0.34 ± 0.14 ^bcd^	0.89 ± 0.25 ^ab^	0.82± 0.40 ^abc^	1.37 ± 0.21 ^a^
17	11.848	1537	L-Aspartic acid, N-(TMS) (2TMS) ester	0.96 ± 0.36	0.80 ± 1.21	0.60 ± 0.08	0.57 ± 0.33	1.24 ± 0.58	1.37 ± 0.57	0.76 ± 0.37	0.38 ± 0.10
18	13.019	1635	Glutamic acid (TMS)	0.79 ± 0.18 ^b^	0.13 ± 0.03 ^b^	0.45 ± 0.13 ^ab^	1.24 ± 0.77 ^b^	0.79 ± 0.39 ^b^	1.17 ± 0.55 ^ab^	0.94 ± 0.58 ^ab^	2.24 ± 0.74 ^a^
	Total amino acids			6.82	2.36	5.40	4.08	5.33	7.39	6.54	8.71
					Sugars (mg/g)						
19	14.739	1789	D-Xylose, tetrakis (TMS)-	0.00 ± 0.00	0.00 ± 0.00	0.00 ± 0.00	0.00 ± 0.00	0.00 ± 0.00	0.00 ± 0.00	0.00 ± 0.00	0.00 ± 0.00
20	15.25	1839	D-Fructose, 1,3,4,5,6-pentakis-O-(TMS)-	0.18 ± 0.03	0.04 ± 0.00	0.20 ± 0.05	0.03 ± 0.03	0.16 ± 0.07	0.01 ± 0.00	0.20 ± 0.26	0.02 ± 0.00
21	15.406	1855	D-Fructose, 1,3,4,5,6-pentakis-O-(TMS)-	14.62 ± 2.21	1.76 ± 0.39	17.55 ± 3.25	1.70 ± 1.01	12.49 ± 4.50	0.16 ± 0.07	27.23 ± 40.44	0.41 ± 0.11
22	15.639	1878	D-Galactofuranose, 1,2,3,5,6-pentakis-O-(TMS)-	6.16 ± 1.05 ^a^	0.45 ± 0.40 ^d^	4.38 ± 1.19 ^d^	0.24 ± 0.18 ^ab^	3.15 ± 1.46 ^bc^	0.02 ± 0.01 ^d^	1.27 ± 0.78 ^cd^	0.14 ± 0.01 ^d^
23	16.064	1920	Galactonic acid, γ-lactone, 4TMS	0.10 ± 0.06 ^a^	0.01 ± 0.01 ^b^	0.06 ± 0.01 ^ab^	0.03 ± 0.03 ^ab^	0.03 ± 0.00 ^b^	0.02 ± 0.01 ^b^	0.02 ± 0.02 ^b^	0.01 ± 0.01 ^b^
24	16.151	1928	D-Glucose, 2,3,4,5,6-pentakis-O-(TMS)-	62.22 ± 3.44 ^a^	13.41 ± 3.58 ^cd^	60.29 ± 4.87 ^cd^	16.69 ± 13.33 ^a^	47.46 ± 10.31 ^ab^	0.18 ± 0.11 ^d^	30.18 ± 14.00 ^bc^	8.67 ± 4.21 ^cd^
25	16.568	1970	D-Mannitol, 1,2,3,4,5,6-hexakis-O-(TMS)-	0.58 ± 0.42	0.09 ± 0.02	0.68 ± 0.28	0.06 ± 0.02	0.26 ± 0.14	0.07 ± 0.04	0.70 ± 0.93	0.07 ± 0.01
26	16.638	1977	D-Glucitol, 6TMS	0.13 ± 0.05	0.01 ± 0.00	0.16 ± 0.13	0.01 ± 0.00	0.12 ± 0.01	0.01 ± 0.00	0.17 ± 0.19	0.01 ± 0.00
27	16.9	2003	D-Allofuranose, pentakis (TMS)	0.02 ± 0.00	0.01 ± 0.00	0.02 ± 0.00	0.00 ± 0.00	0.01 ± 0.00	0.00 ± 0.00	0.00 ± 0.00	0.01 ± 0.00
28	16.997	2014	D-Glucose, 2,3,4,5,6-pentakis-O-(TMS)-	70.11 ± 1.79 ^a^	16.73 ± 4.36 ^cd^	66.43 ± 5.69 ^cd^	19.03 ± 15.56 ^a^	54.74 ± 9.77 ^ab^	0.32 ± 0.20 ^d^	36.94 ± 13.56 ^bc^	9.10 ± 4.15 ^d^
29	17.084	2023	Myo-Inositol, 1,2,3,4,5,6-hexakis-O-(TMS)-	1.28 ± 0.23 ^a^	0.09 ± 0.02 ^bc^	1.23 ± 0.30 ^c^	0.03 ± 0.01 ^a^	0.60 ± 0.34 ^b^	0.14 ± 0.03 ^bc^	0.46 ± 0.20 ^bc^	0.24 ± 0.10 ^bc^
30	18.088	2132	Inositol, 1,2,3,4,5,6-hexakis-O-(TMS)-, scyllo-	1.60 ± 0.23 ^ab^	0.59 ± 0.08 ^abc^	1.50 ± 0.34 ^bc^	0.50 ± 0.32 ^ab^	0.93 ± 0.57 ^abc^	0.22 ± 0.02 ^c^	1.71 ± 0.73 ^a^	0.90 ± 0.40 ^abc^
31	21.478	2538	Unknown sugar	0.02 ± 0.01	0.01 ± 0.01	0.02 ± 0.02	0.02 ± 0.01	0.02 ± 0.01	0.00 ± 0.00	0.01 ± 0.01	0.05 ± 0.05
32	21.917	2593	Sucrose, 8TMS	0.03 ± 0.01 ^a^	0.01 ± 0.00 ^b^	0.01 ± 0.01 ^b^	0.00 ± 0.00 ^b^	0.01 ± 0.00 ^b^	0.00 ± 0.00 ^b^	0.01 ± 0.01 ^b^	0.01 ± 0.00 ^b^
33	22.768	2710	Sucrose, 8TMS	111.27± 4.13 ^a^	5.54 ± 3.86 ^b^	96.90 ± 18.21 ^b^	19.78 ± 6.04 ^a^	85.45 ± 13.76 ^a^	10.78 ± 4.95 ^b^	91.07 ± 19.55 ^a^	11.99 ± 7.27 ^b^
	Total sugars			268.32	38.74	249.43	58.12	205.44	11.94	189.98	31.62
					Free fatty acids (mg/g)						
34	17.33	2050	Palmitic acid, trimethylsilyl ester	1.02 ± 0.16 ^a^	0.41 ± 0.09 ^b^	0.59 ± 0.17 ^b^	0.31 ± 0.10 ^ab^	0.69 ± 0.31 ^ab^	0.42 ± 0.13 ^b^	0.49 ± 0.25 ^b^	0.32 ± 0.06 ^b^
35	18.706	2200	9,12-Octadecadienoic acid (Z,Z)-, trimethylsilyl ester Linoleic acid	3.31 ± 1.42 ^a^	0.58 ± 0.08 ^c^	3.07 ± 0.80 ^bc^	1.11 ± 1.15 ^ab^	0.32 ± 0.04 ^c^	1.22 ± 0.16 ^bc^	1.40 ±0.23 ^abc^	0.72 ± 0.32 ^c^
36	18.909	2224	11-cis-Octadecenoic acid, trimethylsilyl ester Vaccenic acid	0.71 ± 0.15 ^a^	0.06 ± 0.05 ^b^	0.41 ± 0.05 ^b^	0.03 ± 0.00 ^ab^	0.79 ± 0.45 ^a^	0.08 ± 0.02 ^b^	0.30 ± 0.16 ^ab^	0.03 ± 0.01 ^b^
	Total free fatty acids			5.03	1.05	4.06	1.45	1.80	1.72	2.19	1.06

AP-B1-F; Manshiet Al-Ammar bitter apricot fruits (Muddy soil), AP-B1-S; Manshiet Al-Ammar bitter apricot seeds (Muddy soil), AP-B2-F; Al-Amar Al-Kubra bitter apricot fruit (Muddy soil), AP-B2-S; Al-Amar Al-Kubra bitter apricot seed (Muddy soil), AP-M3-F; Nubaria mountain bitter apricot fruit (Sandy soil), AP-M3-S; Nubaria mountain bitter apricot seed (Sandy soil), AP-S2-F; Al-Amar Al-Kubra sweet apricot fruit (Muddy soil), AP-S2-S; Al-Amar Al-Kubra sweet apricot seed (Muddy soil).

**Table 3:** Relative percentage of volatile metabolites in apricot fruits analyzed via GC–MS, *n* = 3. For codes, refer to Table 1. Results are average of 3 independent replicates ± std. deviation. ^a,b^ Different letters in the same line concerning *P. armeniaca* L. specimens indicate significantly different values (*p* < 0.05; Tukey’s test).

No.	Rt. (min.)	KI	Name	AP-B1-F	AP-B2-F	AP-M3-F	AP-S2-F
Alcohols							
1	5.217	774	(Z)-Hex-3-en-1-ol	-	0.07 ± 0.10	-	0.03 ± 0.02
2	8.27	1106	Linalool	0.10 ± 0.08	0.67 ± 0.63	8.86 ± 12.69	0.72 ±0.67
3	8.454	1123	Phenylethyl Alcohol	0.12 ±0.02	0.19 ± 0.10	0.84 ± 0.78	0.20 ± 0.15
4	9.162	1189	Terpinen-4-ol	0.08 ± 0.06	0.22 ± 0.18	0.16 ± 0.08	0.16 ± 0.13
5	9.21	1193	p-Cymen-8-ol	0.36 ± 0.20 ^b^	0.55 ± 0.40 ^a^	0.49 ± 0.19 ^ab^	0.90 ± 0.69 ^ab^
6	9.274	1199	α-Terpineol	9.25± 2.16 ^b^	7.09 ± 2.39 ^ab^	13.27 ± 9.59 ^ab^	6.09 ± 4.51 ^a^
7	9.841	1259	Geraniol	0.03 ± 0.01 ^b^	0.03 ± 0.01 ^a^	0.05 ± 0.06 ^ab^	0.03 ± 0.02 ^a^
8	10.114	1288	1,7-Nonadien-4-ol, 4,8-dimethyl-	1.66 ± 0.79 ^b^	1.57 ±1.20 ^a^	0.46 ± 0.15 ^a^	2.91 ± 2.39 ^a^
9	10.32	1309	p-Menth-1-en-9-ol	-	-	0.32 ± 0.51	0.03 ± 0.03
Total alcohols				11.6	10.39	24.45	11.07
Ketones							
10	7.805	1068	Acetophenone	0.061 ± 0.05	0.17 ± 0.12	0.09 ± 0.02	0.26 ± 0.24
11	7.87	1070	Isophorone	0.20 ± 0.16	1.71 ± 1.45	0.19 ± 0.19	2.46 ± 2.13
12	9.788	1254.48	Pulegone	0.01 ± 0.00	0.05 ± 0.04	0.10 ± 0.07	0.02 ± 0.02
13	11.124	1395	Anisketone	1.57 ± 0.79	0.30 ± 0.32	0.37 ± 0.23	3.76 ± 6.11
14	11.272	1410	(Z)-Jasmone	0.02 ± 0.00	0.05 ± 0.02	0.02 ± 0.00	0.04 ± 0.03
15	11.683	1450	Geranyl acetone	2.68 ± 0.68 ^a^	1.99 ± 1.23 ^b^	1.84 ± 0.55 ^ab^	3.61 ± 2.95 ^b^
16	14.06	1692	Turmerone	0.24 ± 0.01 ^a^	0.17 ± 0.11 ^b^	0.32 ± 0.22 ^ab^	0.12 ± 0.05 ^b^
Total ketones				4.78	4.44	2.93	10.27
Aldehydes							
17	8.034	1085	5-formylfurfural	0.33 ± 0.13	0.31 ± 0.50	0.38 ± 0.36	0.14 ± 0.06
18	9.121	1185	Benzaldehyde, 2,5-dimethyl-	0.16 ± 0.05 ^b^	0.22 ± 0.16 ^a^	0.21 ± 0.13 ^ab^	0.25 ± 0.21 ^a^
19	9.365	1209	n-Decanal	0.12 ± 0.03	0.11 ± 0.00	0.24 ± 0.20	0.08 ± 0.06
20	9.4	1212	Safranal	0.05 ± 0.03	0.05 ± 0.04	0.21 ± 0.03	0.14 ± 0.12
21	9.566	1229	5-Hydroxymethylfurfural	2.71 ± 1.23 ^b^	0.87 ± 1.49 ^ab^	1.71 ± 1.47 ^ab^	0.82 ± 0.42 ^a^
22	9.613	1235	β-Cyclocitral	1.71 ± 1.30 ^b^	1.45 ± 1.19 ^a^	0.46 ± 0.28 ^a^	2.39 ± 2.06 ^a^
23	9.93	1268	p-Anisaldehyde	1.53 ± 0.48	1.76 ± 0.59	4.66 ± 4.56	7.08 ± 8.85
24	10.026	1279	α-Citral	0.17 ± 0.03 ^b^	0.18 ± 0.08 ^a^	0.30 ± 0.17 ^ab^	0.34 ± 0.04 ^a^
25	10.09	1285	(E)-Cinnamaldehyde	1.00 ± 0.46	0.35 ± 0.51	0.50 ± 0.40	0.79 ± 1.15
26	10.707	1351	Piperonal	0.10 ± 0.05	0.08 ± 0.07	0.07 ± 0.06	0.05 ± 0.09
27	11.263	1409	Methyleugenol	0.26 ± 0.09 ^b^	0.18 ± 0.10 ^a^	0.05 ± 0.03 ^a^	0.24 ± 0.19 ^a^
28	13.008	1579	trans-4-Methoxycinnamaldehyde	0.09 ± 0.05	0.02 ± 0.03	0.02	0.41
Total Aldehyde				8.23	5.58	8.81	12.73
Aromatics							
29	8.2	1100	p-Cymenene	0.03 ± 0.02	0.07 ± 0.03	0.05 ± 0.06	0.10 ± 0.09
30	8.309	1110	Unknown	0.12 ± 0.05	0.94 ± 0.76	0.31 ± 0.23	0.66 ± 0.51
31	10.372	1315	α-Methylnaphthalene	0.15 ± 0.02	0.18 ± 0.03	0.38 ± 0.27	0.12 ± 0.04
32	10.541	1333	β-Methylnaphthalene	1.92 ± 3.00	0.02 ± 0.02	0.03 ± 0.02	0.22 ± 0.19
33	11.33	1415	Diphenyl ether	0.20 ± 0.07	0.13 ± 0.19	0.20 ± 0.15	0.10 ± 0.15
34	11.531	1435	Dimethylnaphthalene	0.14 ± 0.04	0.06 ± 0.03	0.03 ± 0.02	0.13
35	11.945	1475	Unknown	0.22 ± 0.11	0.10 ± 0.17	0.05 ± 0.06	0.02 ± 0.05
Total aromatics				2.78	1.5	1.05	1.35
Esters							
36	8.26	1105	Methyl benzoate	2.02 ± 1.37	1.58 ± 0.69	2.22 ± 0.81	0.23
37	8.971	1171	Benzyl acetate	0.21 ± 0.16	0.13 ± 0.06	0.07 ± 0.08	0.19
38	9.341	1205	Methyl salicylate	0.11 ± 0.02	0.09 ± 0.03	0.09 ± 0.06	0.07
39	10.804	1361	α-Terpinyl acetate	0.08 ± 0.04 ^a^	0.01 ± 0.01 ^b^	0.11 ± 0.08 ^ab^	0.11 ^ab^
40	14.901	1785	Benzyl Benzoate	0.25 ± 0.11	0.40 ± 0.29	0.19 ± 0.12	0.35
Total esters				2.67	2.21	2.68	0.95
Ethers/Oxides							
41	9.349	1207	Estragole	0.24 ± 0.12	0.17 ± 0.10	0.61 ± 0.46	0.28 ± 0.16
42	9.889	1264	Anethole	0.02 ± 0.00	0.09 ± 0.06	0.10 ± 0.05	0.13 ± 0.14
43	10.215	1299	Anethole isomer	6.02 ± 2.57	2.57 ± 3.02	4.53 ± 3.30	19.85 ± 32.11
44	10.266	1304	Safrole	0.07 ± 0.02	0.02 ± 0.00	0.16 ± 0.12	0.06 ± 0.04
45	10.882	1370	Eugenol	0.11 ± 0.02	0.02 ± 0.01	0.37 ± 0.02	0.15 ± 0.11
46	8.864	1161	Nerol oxide	0.77 ± 0.11	0.01 ± 0.00	0.26 ± 0.42	0.43 ± 0.28
Total ethers/Oxides				7.23	2.88	6.03	20.9
Ionones							
47	9.951	1271	α-Ionene	0.047 ± 0.03	0.03 ± 0.02	0.42 ± 0.27	0.41 ± 0.38
48	10.913	1373	Dehydro-ar-ionene	0.65 ± 0.18	0.19 ± 0.07	2.35 ± 1.52	0.31 ± 0.27
49	10.942	1376	Megastigma-4,6(Z),8(E)-triene	0.03 ± 0.02	0.04 ±0.03	0.09 ± 0.05	0.05 ± 0.04
50	11.633	1445	Dihydro-β-ionone	0.02 ± 0.00	-	0.26 ± 0.17	0.08 ± 0.05
51	12.096	1490.73	β-Ionone	13.75 ± 3.37 ^a^	6.09 ± 0.29 ^b^	4.68 ± 3.40 ^b^	11.52 ± 8.80 ^b^
Total ionones				14.49	6.35	7.80	12.37
Acids							
52	10.8	1361	n-Decanoic acid	0.06 ± 0.01	0.48 ± 0.64	0.07 ± 0.04	0.37 ± 0.31
Total acids				0.06	0.48	0.07	0.37
Nitrogenous Compounds							
53	11.379	1420.76	Methyl methanthranilate	0.11 ± 0.02	0.05 ± 0.00	0.31 ± 0.10	0.08 ± 0.05
Total Nitrogenous Compounds				0.11	0.05	0.31	0.08
Phenols							
54	11.737	1455.71	Thymoquinol	0.67	1.00	0.76	0.27
Total phenols				0.67	1.00	0.76	0.27
Aliphatic hydrocarbon							
55	11.17	1400	Tetradecane	0.32 ± 0.21 ^b^	0.33 ± 0.24 ^a^	0.22 ± 0.20 ^ab^	0.19 ± 0.15 ^a^
56	13.219	1599	Hexadecane	0.06 ± 0.02	0.06 ± 0.04	0.02 ± 0.02	0.07 ± 0.03
57	14.244	1712	Heptadecane	0.10 ± 0.02	0.23 ± 0.18	0.04 ± 0.06	0.21± 0.14
58	15.195	1816	Octadecane	8.33 ± 14.41	0.14 ± 0.09	0.06 ± 0.04	0.11 ± 0.07
59	15.904	1891	Nonadecane	0.14 ± 0.04	0.32 ± 0.07	0.11 ± 0.03	0.25 ± 0.09
Total aliphatic hydrocarbon				8.95	1.08	0.45	0.83
Lactones							
60	11.88	1469	γ-Decanolactone	17.14 ± 4.60	26.34 ± 6.66	31.19 ± 12.99	5.00 ± 4.37
61	12.179	1498	δ-Decalactone	2.16 ± 0.55	2.73 ± 0.16	2.51 ± 1.32	0.15 ± 0.13
62	12.698	1549	Dihydroactinidiolide	5.54 ± 1.16 ^a^	7.76 ± 2.45 ^ab^	1.46 ± 0.68 ^b^	9.13 ± 6.31 ^ab^
63	14.156	1703	γ-Dodecalactone	0.61 ± 0.13	1.48 ± 0.32	1.98 ± 0.72	0.54 ± 0.369
Total lactones				24.45	38.31	37.14	14.82
Fatty acids/esters							
64	12.345	1515	Methyl laurate	0.75 ± 0.32	0.70 ± 0.34	0.23 ± 0.11	0.14 ± 0.12
65	14.439	1734	Methyl myristate	1.20 ± 0.47	2.13 ± 0.97	0.68 ± 0.23	0.34 ± 0.26
66	14.676	1761	Myristic acid	0.05 ± 0.01	0.11 ± 0.08	0.05 ± 0.03	0.32 ± 0.20
67	15.956	1896	Methyl palmitoleate	0.10 ± 0.04 ^a^	0.34 ± 0.27 ^b^	0.08 ± 0.03 ^ab^	0.08 ± 0.06 ^ab^
68	16.164	1918	Methyl palmitate	9.42 ± 3.29 ^a^	20.32 ± 8.86 ^b^	5.80 ± 1.90 ^b^	8.10 ± 6.70 ^b^
69	16.471	1950	Palmitic acid	0.31 ± 0.04 ^a^	0.68 ± 0.93 ^ab^	0.13 ± 0.07 ^b^	2.77 ± 2.04 ^b^
70	18.234	2134	8,11-Octadecadienoic acid, methyl ester	0.76 ± 0.32	1.11 ± 0.33	0.22 ± 0.06	1.88 ± 1.90
Total fatty acids/ester				12.59	25.39	7.19	13.63

AP-B1-F; Manshiet Al-Ammar bitter apricot fruits (Muddy soil), AP-B2-F; Al-Amar Al-Kubra bitter apricot fruit (Muddy soil), AP-M3-F; Nubaria mountain bitter apricot fruit (Sandy soil), AP-S2-F; Al-Amar Al-Kubra sweet apricot fruit (Muddy soil).

## Data Availability

The data presented in this study are available on request from the corresponding author.

## Supplementary Materials

The following supporting information can be downloaded at: https://www.mdpi.com/article/10.3390/foods11091339/s1, Figure S1: Principal component analyses of silylated primary metabolites in seed extracts as analyzed by GC-MS (*n* = 3). (A) Score plot of PC1 vs. PC2 scores. (B) Loading plot for PC1 and PC2 contributing mass peaks and their assignments. The metabolome clusters are placed in two dimensional space at the distinct locations defined by two vectors of principal component PC1 = 64% and PC2 = 18%.; Figure S2: Principal component analyses of silylated fruit of Apricot methanol extracts as analyzed by GC-MS (*n* = 3). (A) Score plot of PC1 vs. PC2 scores. (B) Loading plot for PC1 and PC2 contributing mass peaks and their assignments. The metabolome clusters are placed in two dimensional space at the distinct locations defined by two vectors of principal component PC1 = 62% and PC2 = 18%; Figure S3: Unsupervised multivariate data analyses of the studied apricot fruit cultivars derived from modeling volatile profiles analyzed via GC–MS (*n* = 3). (A) PCA score plot of PC1 vs. PC2 scores. (B) The respective loading plot for PC1 and PC2, providing their assignments. The metabolome clusters are placed in two-dimensional space at the distinct locations defined by two vectors of principal component PC1 = 34% and PC2 = 23%. (C) HCA plot; Figure S4: GC–MS-based OPLS-DA score plot (A) derived from modeling volatiles of sweet apricot fruit cultivar versus all bitter apricot fruit cultivars (*n* = 3). The respective loading S-plots (B) showing the covariance *p* (1) against the correlation *p*(cor) (1) of the variables of the discriminating component of the OPLS-DA model. Cut-off values of *p* = 0.02 were used. Designated variables are highlighted and identifications are discussed in the text.

## References

[1] Farag M.A., El Senousy A.S., El-Ahmady S.H., Porzel A., Wessjohann L.A. (2019). Comparative Metabolome-Based Classification of Senna Drugs: A Prospect for Phyto-Equivalency of Its Different Commercial Products. Metabolomics.

[2] Ramadan N.S., Wessjohann L.A., Mocan A., Vodnar D.C., ElSayed N.H., ElToumy S.A., Mohamed D.A., Aziz Z.A., Ehrlich A., Farag M.A. (2020). Nutrient and Sensory Metabolites Profiling of *Averrhoa carambola* L. (Starfruit) in the Context of Its Origin and Ripening Stage by GC/MS and Chemometric Analysis. Molecules.

[3] Mesarović J., Trifković J., Tosti T., Fotirić Akšić M., Milatović D., Ličina V., Milojković-Opsenica D. (2018). Relationship between Ripening Time and Sugar Content of Apricot (*Prunus armeniaca* L.) Kernels. Acta Physiol. Plant..

[4] Jiang F., Zhang J., Wang S., Yang L., Luo Y., Gao S., Zhang M., Wu S., Hu S., Sun H. (2019). The Apricot (*Prunus armeniaca* L.) Genome Elucidates Rosaceae Evolution and Beta-Carotenoid Synthesis. Hortic. Res..

[5] Kovacikova E., Kovacik A., Halenar M., Tokarova K., Chrastinova L., Ondruska L., Jurcik R., Kolesar E., Valuch J., Kolesarova A. (2019). Potential Toxicity of Cyanogenic Glycoside Amygdalin and Bitter Apricot Seed in Rabbits—Health Status Evaluation. J. Anim. Physiol. Anim. Nutr..

[6] Yiǧit D., Yiǧit N., Mavi A. (2009). Antioxidant and Antimicrobial Activities of Bitter and Sweet Apricot (*Prunus armeniaca* L.) Kernels. Braz. J. Med. Biol. Res..

[7] Wani S.M., Jan N., Wani T.A., Ahmad M., Masoodi F.A., Gani A. (2017). Optimization of Antioxidant Activity and Total Polyphenols of Dried Apricot Fruit Extracts (*Prunus armeniaca* L.) Using Response Surface Methodology. J. Saudi Soc. Agric. Sci..

[8] Raafat K., El-Darra N., Saleh F.A., Rajha H.N., Maroun R.G., Louka N. (2018). Infrared-Assisted Extraction and HPLC-Analysis of *Prunus armeniaca* L. Pomace and Detoxified-Kernel and Their Antidiabetic Effects. Phytochem. Anal..

[9] Merzouki A., Ed-derfoufi F., Molero Mesa J. (2000). Contribution to the Knowledge of Rifian Traditional Medicine. II: Folk Medicine in Ksar Lakbir District (NW Morocco). Fitoterapia.

[10] FAOSTAT. https://www.fao.org/faostat/en/#home.

[11] Awad N., Gabr M., Gawish M. (2019). Morphological Evaluation and Genetic Identification of Some Local Apricot Lines. J. Plant Prod..

[12] Zohry A.E.H., Ouda S., Hamd-Alla W., Shalaby E.S. (2017). Evaluation of Different Crop Sequences for Wheat and Maize in Sandy Soil. Acta Agric. Slov..

[13] Katkat A.V. (2009). Effects of Soil and Foliar Applications of Humic Substances on Dry Weight and Mineral Nutrients Uptake of Wheat under Calcareous Soil Conditions. Aust. J. Basic Appl. Sci..

[14] Usowicz B., Lipiec J. (2017). Spatial Variability of Soil Properties and Cereal Yield in a Cultivated Field on Sandy Soil. Soil Tillage Res..

[15] Liu W., Liu N., Zhang Y., Yu X., Sun M., Xu M., Zhang Q., Liu S. (2012). Kernel-Using Apricot Resources and Its Utilization. Acta Hortic..

[16] Ighbareyeh J.M.H., Carmona E.C., Mohammed M.H., Suliemieh A.A.A. (2016). Study of Biology and Bioclimatology Applied of Apricot (*Prunus armeniaca* L.): To Increase the Economy and Maintaining Food Security in Palestine. Int. J. Res. Stud. Biosci..

[17] Negri P., Bassi D., Magnanini E., Rizzo M., Bartolozzi F. (2008). Bitterness Inheritance in Apricot (*P. armeniaca* L.) Seeds. Tree Genet. Genomes.

[18] Tousson E., Hafez E., Abo Gazia M.M., Salem S.B., Mutar T.F. (2020). Hepatic Ameliorative Role of Vitamin B17 against Ehrlich Ascites Carcinoma–Induced Liver Toxicity. Environ. Sci. Pollut. Res..

[19] Melgarejo P., Calín-Sánchez Á., Carbonell-Barrachina Á.A., Martínez-Nicolás J.J., Legua P., Martínez R., Hernández F. (2014). Antioxidant Activity, Volatile Composition and Sensory Profile of Four New Very-Early Apricots (*Prunus armeniaca* L.). J. Sci. Food Agric..

[20] Schmitzer V., Slatnar A., Mikulic-Petkovsek M., Veberic R., Krska B., Stampar F. (2011). Comparative Study of Primary and Secondary Metabolites in Apricot (*Prunus armeniaca* L.) Cultivars. J. Sci. Food Agric..

[21] Solis-Solis H.M., Calderon-Santoyo M., Gutierrez-Martinez P., Schorr-Galindo S., Ragazzo-Sanchez J.A. (2007). Discrimination of Eight Varieties of Apricot (*Prunus armeniaca*) by Electronic Nose, LLE and SPME Using GC-MS and Multivariate Analysis. Sens. Actuators B Chem..

[22] Stryjecka M., Kiełtyka-Dadasiewicz A., Michalak M., Rachoń L., Głowacka A. (2019). Chemical Composition and Antioxidant Properties of Oils from the Seeds of Five Apricot (*Prunus armeniaca* L.) Cultivars. J. Oleo Sci..

[23] Zhang H., Liu W., Fang J., Chen S., Liu Y., Wu B., Li S. (2014). Volatile Profiles of Apricot Cultivars (*Prunus armeniaca* Lam.) Evaluated by Head Space Solid Phase Microextraction Gas Chromatography Mass Spectrometry. Anal. Lett..

[24] Bhanger M.I., Anwar F., Memon N., Qadir R. (2020). Cold Pressed Apricot (*Prunus armeniaca* L.) Kernel Oil. Cold Pressed Oils.

[25] Luber F., Demmel A., Hosken A., Busch U., Engel K.H. (2012). Apricot DNA as an Indicator for Persipan: Detection and Quantitation in Marzipan Using Ligation-Dependent Probe Amplification. J. Agric. Food Chem..

[26] Farag M.A., Khattab A.R., Shamma S., Afifi S.M. (2021). Profiling of Primary Metabolites and Volatile Determinants in Mahlab Cherry (*Prunus mahaleb* L.) Seeds in the Context of Its Different Varieties and Roasting as Analyzed Using Chemometric Tools. Foods.

[27] Fan X., Zhao H., Wang X., Cao J., Jiang W. (2017). Sugar and Organic Acid Composition of Apricot and Their Contribution to Sensory Quality and Consumer Satisfaction. Sci. Hortic..

[28] Bae H., Yun S.K., Yoon I.K., Nam E.Y., Kwon J.H., Jun J.H. (2014). Assessment of Organic Acid and Sugar Composition in Apricot, Plumcot, Plum, and Peach during Fruit Development. J. Appl. Bot. Food Qual..

[29] Caliskan O., Bayazit S., Sumbul A. (2012). Fruit Quality and Phytochemical Attributes of Some Apricot (*Prunus armeniaca* L.) Cultivars as Affected by Genotypes and Seasons. Not. Bot. Horti Agrobot. Cluj-Napoca.

[30] Farag M.A., El-Kersh D.M., Ehrlich A., Choucry M.A., El-Seedi H., Frolov A., Wessjohann L.A. (2019). Variation in *Ceratonia siliqua* Pod Metabolome in Context of Its Different Geographical Origin, Ripening Stage and Roasting Process. Food Chem..

[31] Naryal A., Acharya S., Bhardwaj A.K., Kant A., Chaurasia O.P., Stobdan T. (2019). Altitudinal Effect on Sugar Contents and Sugar Profiles in Dried Apricot (*Prunus armeniaca* L.) Fruit. J. Food Compos. Anal..

[32] Xi W., Zheng H., Zhang Q., Li W. (2016). Profiling Taste and Aroma Compound Metabolism during Apricot Fruit Development and Ripening. Int. J. Mol. Sci..

[33] Milošević T., Milošević N., Glišić I., Mladenović J. (2012). Fruit Quality, Phenolics Content and Antioxidant Capacity of New Apricot Cultivars from Serbia. Acta Sci. Pol. Hortorum Cultus.

[34] Gill S.K., Lever E., Emery P.W., Whelan K. (2019). Nutrient, Fibre, Sorbitol and Chlorogenic Acid Content of Prunes (*Prunus domestica*): An Updated Analysis and Comparison of Different Countries of Origin and Database Values. Int. J. Food Sci. Nutr..

[35] Dine Tariq Bouhlali E., Derouich M., Meziani R., Bourkhis B., Filali-Zegzouti Y., Alem C. (2020). Nutritional, Mineral and Organic Acid Composition of Syrups Produced from Six Moroccan Date Fruit (*Phoenix dactylifera* L.) Varieties. J. Food Compos. Anal..

[36] Choe U., Sun J., Bailoni E., Chen P., Li Y., Gao B., Wang T.T.Y., Rao J., Yu L.L. (2021). Chemical Composition of Tomato Seed Flours, and Their Radical Scavenging, Anti-Inflammatory and Gut Microbiota Modulating Properties. Molecules.

[37] Nazir N., Khalil A.A.K., Nisar M., Zahoor M., Ahmad S. (2020). HPLC-UV Characterization, Anticholinesterase, and Free Radical-Scavenging Activities of *Rosa moschata* Herrm. Leaves and Fruits Methanolic Extracts. Braz. J. Bot..

[38] Świątkowski M., Lanka S., Czylkowska A., Gas K., Sawicki M. (2021). Structural, Spectroscopic, Thermal, and Magnetic Properties of a New Dinuclear Copper Coordination Compound with Tiglic Acid. Materials.

[39] Tomás-Barberán F.A., Ruiz D., Valero D., Rivera D., Obón C., Sánchez-Roca C., Gil M.I. (2013). Health Benefits from *Pomegranates* and Stone Fruit, Including Plums, Peaches, Apricots and Cherries. Bioact. Fruit Health Benefits Funct. Foods.

[40] Gurrieri F., Audergon J.-M., Albagnac G., Reich M. (2001). Soluble Sugars and Carboxylic Acids in Ripe Apricot Fruit as Parameters for Distinguishing Different Cultivars. Euphytica.

[41] Ibrahim N., Taleb M., Heiss A.G., Kropf M., Farag M.A. (2021). GC-MS Based Metabolites Profiling of Nutrients and Anti-Nutrients in 10 Lathyrus Seed Genotypes: A Prospect for Phyto-Equivalency and Chemotaxonomy. Food Biosci..

[42] Field C.J., Blewett H.H., Proctor S., Vine D. (2009). Human Health Benefits of Vaccenic Acid. Appl. Physiol. Nutr. Metab..

[43] Aumeistere L., Beluško A., Ciproviča I., Zavadska D. (2021). Trans Fatty Acids in Human Milk in Latvia: Association with Dietary Habits during the Lactation Period. Nutrients.

[44] Hrichi S., Rigano F., Chaabane-Banaoues R., Oulad El Majdoub Y., Mangraviti D., Di Marco D., Babba H., Dugo P., Mondello L., Mighri Z. (2020). Identification of Fatty Acid, Lipid and Polyphenol Compounds from *Prunus armeniaca* L. Kernel Extracts. Foods.

[45] Farag M.A., Afifi S.M., Rasheed D.M., Khattab A.R. (2021). Revealing Compositional Attributes of Glossostemon Bruguieri Desf. Root Geographic Origin and Roasting Impact via Chemometric Modeling of SPME-GC-MS and NMR Metabolite Profiles. J. Food Compos. Anal..

[46] Azodanlou R., Darbellay C., Luisier J.-L., Villettaz J.-C., Amadò R. (2003). Development of a Model for Quality Assessment of Tomatoes and Apricots. LWT-Food Sci. Technol..

[47] Lo Bianco R., Farina V., Indelicato S.G., Filizzola F., Agozzino P. (2010). Fruit Physical, Chemical and Aromatic Attributes of Early, Intermediate and Late Apricot Cultivars. J. Sci. Food Agric..

[48] Pintea A., Dulf F.V., Bunea A., Socaci S.A., Pop E.A., Opriță V.-A., Giuffrida D., Cacciola F., Bartolomeo G., Mondello L. (2020). Carotenoids, Fatty Acids, and Volatile Compounds in Apricot Cultivars from Romania—A Chemometric Approach. Antioxidants.

[49] Guillot S., Peytavi L., Bureau S., Boulanger R., Lepoutre J.P., Crouzet J., Schorr-Galindo S. (2006). Aroma Characterization of Various Apricot Varieties Using Headspace-Solid Phase Microextraction Combined with Gas Chromatography-Mass Spectrometry and Gas Chromatography-Olfactometry. Food Chem..

[50] Sayed-Ahmad B., Talou T., Saad Z., Hijazi A., Merah O. (2017). The Apiaceae: Ethnomedicinal Family as Source for Industrial Uses. Ind. Crops Prod..

[51] Khalil M.N.A., Fekry M.I., Farag M.A. (2017). Metabolome Based Volatiles Profiling in 13 Date Palm Fruit Varieties from Egypt via SPME GC–MS and Chemometrics. Food Chem..

[52] Göğüş F., Özel M.Z., Lewis A.C. (2007). The Effect of Various Drying Techniques on Apricot Volatiles Analysed Using Direct Thermal Desorption-GC–TOF/MS. Talanta.

