# Supplementary material for: Profiling of Primary Metabolites and Volatiles in Apricot (Prunus armeniaca L.) Seed Kernels and Fruits in the Context of Its Different Cultivars and Soil Type as Analyzed Using Chemometric Tools
Source: Foods. 2022 May 4;11(9):1339. doi: 10.3390/foods11091339 (PMC9104916; doi:10.3390/foods11091339)
Supplement: Supplementary file 1 [file foods-11-01339-s001.zip › foods-1675396-supplementary.pdf]

Supplementary Figures

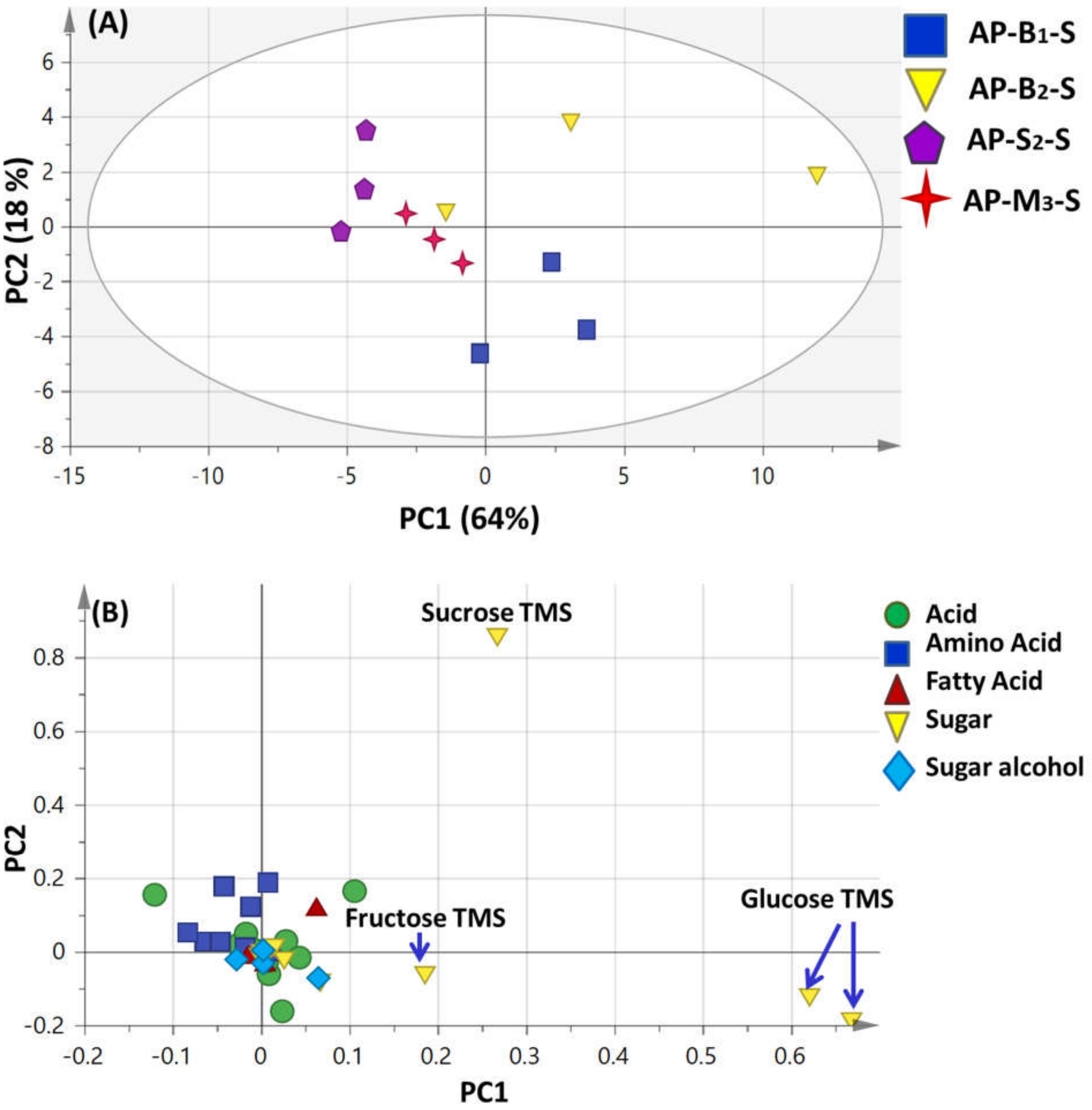

Figure S1: Principal component analyses of silylated primary metabolites in seed extracts as analyzed by GC-MS (n = 3). (**A**) Score plot of PC1 vs. PC2 scores. (**B**) Loading plot for PC1 and PC2 contributing mass peaks and their assignments. The metabolome clusters are placed in two dimensional space at the distinct locations defined by two vectors of principal component PC1 = 64% and PC2 = 18%.

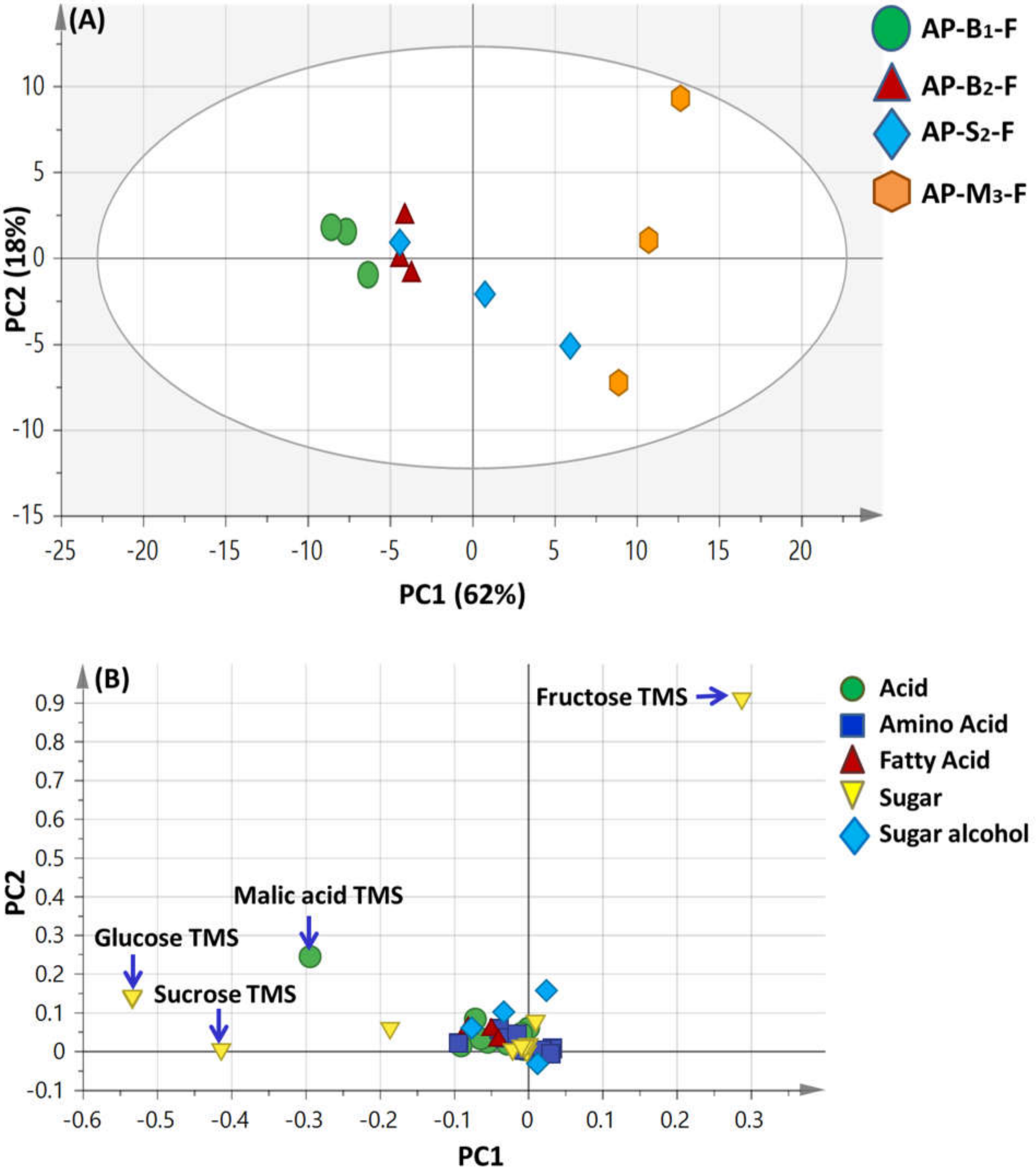

Figure S2: Principal component analyses of silylated fruit of Apricot methanol extracts as analyzed by GC-MS (n = 3). (**A**) Score plot of PC1 vs. PC2 scores. (**B**) Loading plot for PC1 and PC2 contributing mass peaks and their assignments. The metabolome clusters are placed in two dimensional space at the distinct locations defined by two vectors of principal component PC1 = 62% and PC2 = 18%.

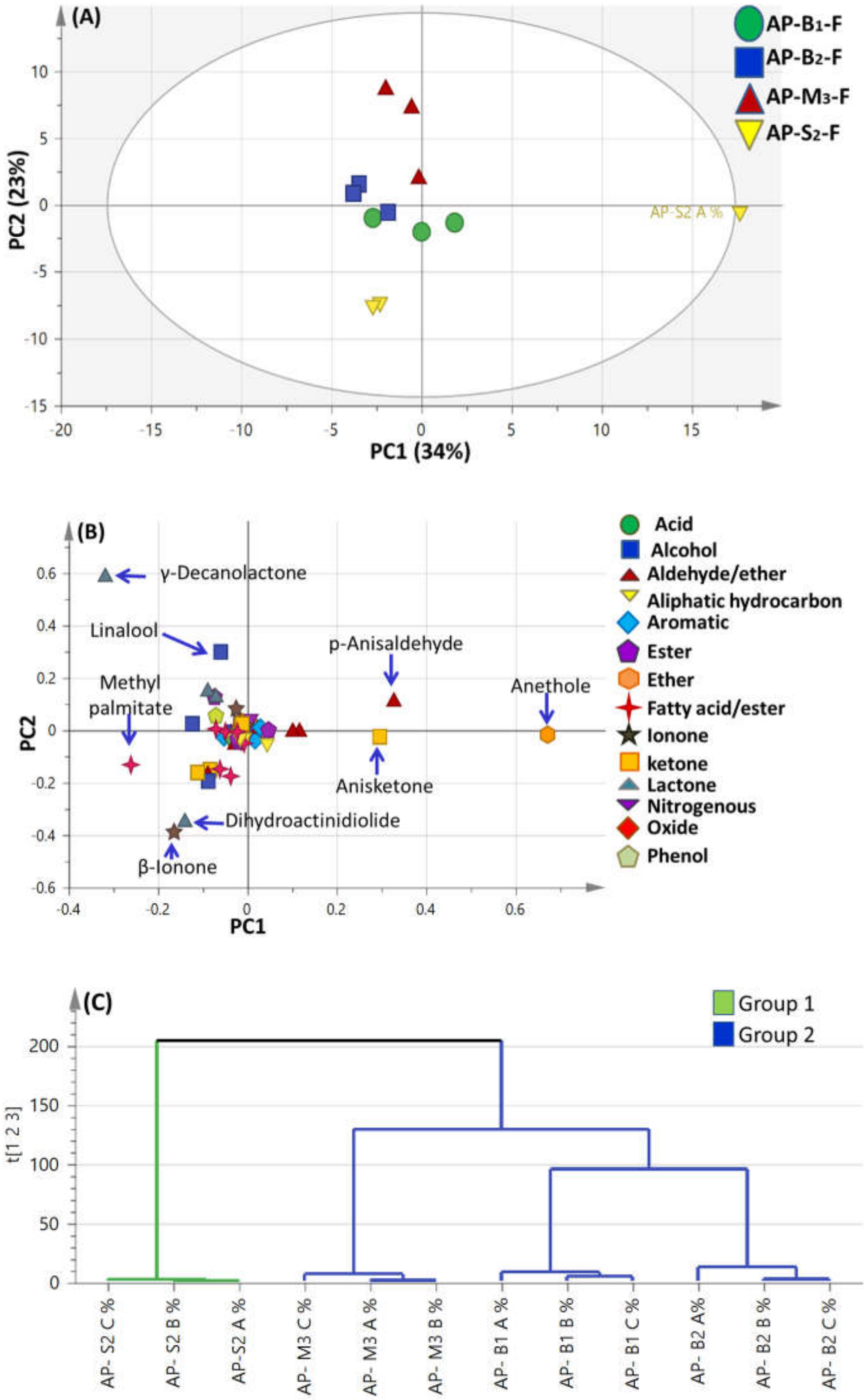

Figure S3: Unsupervised multivariate data analyses of the studied apricot fruit cultivars derived from modeling volatile profiles analyzed via GC–MS (n = 3). (**A**) PCA score plot of PC1 vs. PC2 scores. (**B**) The respective loading plot for PC1 and PC2, providing their assignments. The metabolome clusters are placed in two-dimensional space at the distinct locations defined by two vectors of principal component PC1 = 34% and PC2 = 23%. (**C**) HCA plot.

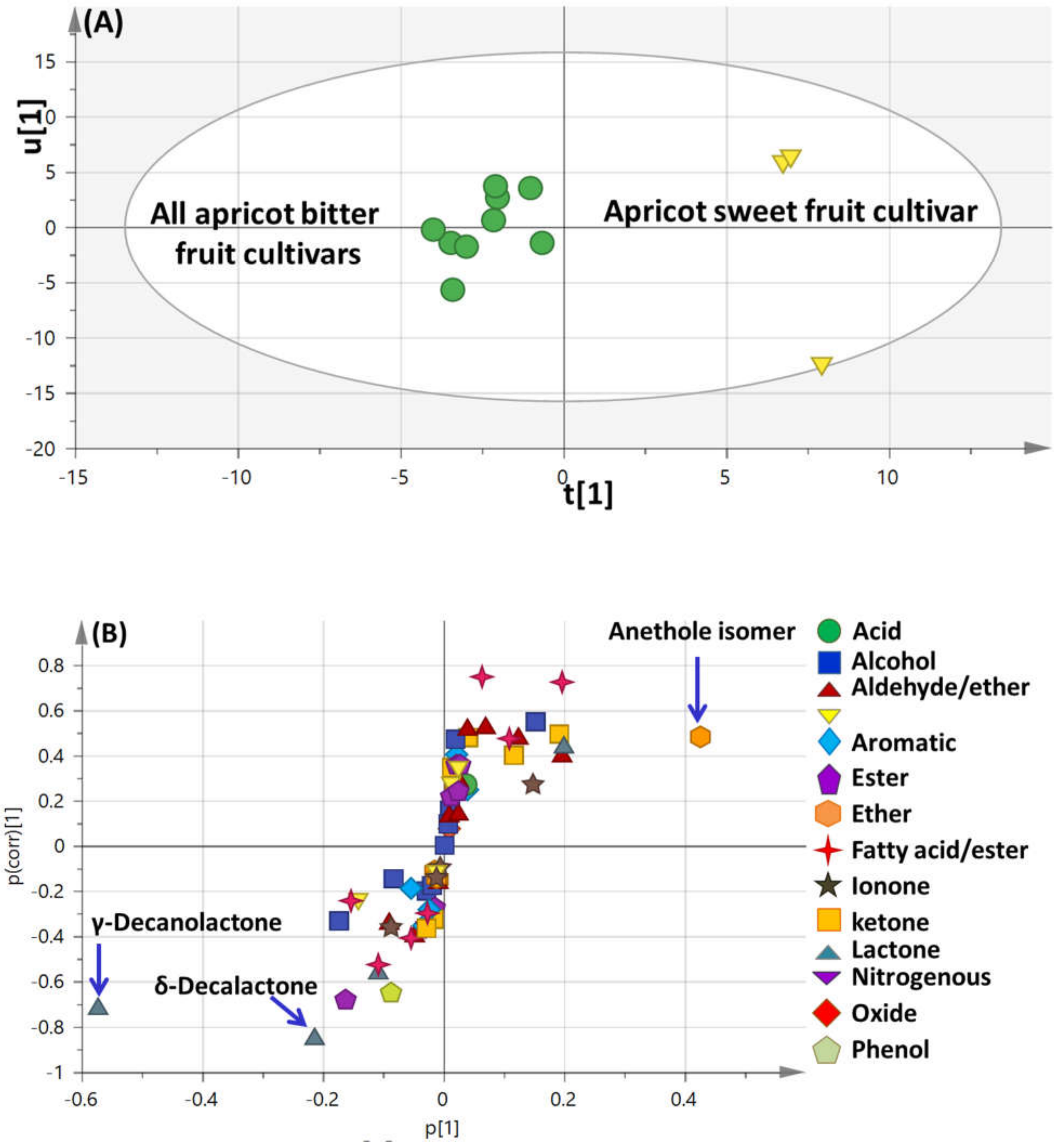

Figure S4: GC–MS-based OPLS-DA score plot (**A**) derived from modeling volatiles of sweet apricot fruit cultivar versus all bitter apricot fruit cultivars (n = 3). The respective loading S-plots (**B)** showing the covariance p [1] against the correlation p(cor) [1] of the variables of the discriminating component of the OPLS-DA model. Cut-off values of p =0.02 were used. Designated variables are highlighted and identifications are discussed in the text.
